# SARS-CoV-2 Portrayed against HIV: Contrary Viral Strategies in Similar Disguise

**DOI:** 10.3390/microorganisms9071389

**Published:** 2021-06-27

**Authors:** Ralf Duerr, Keaton M. Crosse, Ana M. Valero-Jimenez, Meike Dittmann

**Affiliations:** Department of Microbiology, New York University School of Medicine, New York, NY 10016, USA; Keaton.Crosse@nyulangone.org (K.M.C.); AnaMayela.ValeroJimenez@nyulangone.org (A.M.V.-J.); Meike.Dittmann@nyulangone.org (M.D.)

**Keywords:** SARS-CoV-2, HIV, zoonotic viruses, COVID-19 and AIDS pandemics, viral entry

## Abstract

SARS-CoV-2 and HIV are zoonotic viruses that rapidly reached pandemic scale, causing global losses and fear. The COVID-19 and AIDS pandemics ignited massive efforts worldwide to develop antiviral strategies and characterize viral architectures, biological and immunological properties, and clinical outcomes. Although both viruses have a comparable appearance as enveloped viruses with positive-stranded RNA and envelope spikes mediating cellular entry, the entry process, downstream biological and immunological pathways, clinical outcomes, and disease courses are strikingly different. This review provides a systemic comparison of both viruses’ structural and functional characteristics, delineating their distinct strategies for efficient spread.

## 1. Introduction

SARS-CoV-2 and HIV each rapidly became and continue to be considerable global health concerns. Unparalleled scientific efforts enabled characterization of these viruses and their resulting diseases in record time, which has led to rapid development of public health measures and antiviral strategies. Both viruses have elementary similarities, being enveloped viruses with a positive (+) single-stranded (ss) RNA genome. Consequently, preventive and therapeutic approaches that were studied or established for HIV have been tested against SARS-CoV-2 including vaccination strategies, reverse vaccinology, monoclonal antibodies (mAbs), and investigational or approved anti-HIV drugs [1,2,3]. While more data keep unfolding, we know that despite overall similarities, both viruses have key differences spanning structural and functional characteristics, cellular tropism, induced immune responses, clinical outcome, and responsiveness to vaccines and treatments (Table 1). For SARS-CoV-2, emergency use authorizations of vaccines have been achieved to prevent severe coronavirus disease COVID-19; however, treatment options remain scarce. For HIV, more than 45 antiretroviral drugs are available to manage chronic disease and delay AIDS; however, we still lack an efficient vaccine. This review illustrates the fundamental similarities and differences of SARS-CoV-2 and HIV to further our understanding of their biological and clinical characteristics and thus support the development of antiviral strategies against current and future viral outbreaks. Since HIV-1 is responsible for >95% of global HIV infections and HIV-2 has remained largely restricted to Western Africa [4], this review focuses on HIV-1.

## 2. Methods

Structural analyses were performed using UCSF Chimera v.1.15rc [88] based on pdb files downloaded from the Research Collaboratory for Structural Bioinformatics Protein Data Bank (RCSB PDB). Protein structures were generated using pdb files 3j5m or 6wpu (prefusion “closed” HIV-1 Env), S.pdb [89] (prefusion “closed” SARS-CoV-2 spike trimer), 4l1a (HIV-1 protease in complex with lopinavir), 6wnp (SARS-CoV-2 main protease in complex with Boceprevir), 3v4i/3v81 (HIV-1 reverse transcriptase [RT] in complex with DNA, azathioprine-triphosphate [3v4i; red], and nevirapine [3v81; purple], the latter superimposed after structural overlay of 3v4i and 3v81 RT structures), and 7bv2 (SARS-CoV-2 polymerase RdRp [NSP12] in complex with NSP7, NSP8, template-primer RNA, and Remdesivir triphosphate). Complex entry models were created using structural overlays using MatchMaker in Chimera of pdb structures 6vyb, 6m17, and S_ACE2 [89] (SARS-CoV-2), or 6met and 5vn3 (HIV-1). Models of fusion intermediates were created based on pdb files 6m3w (SARS-CoV-2) and 2zfc (HIV-1). In silico glycosylation of proteins was performed using GlyProt [90], and the composition of oligomannose, hybrid, and complex N-glycans matched with reference literature [91,92]. Viral life cycle schematics were created with BioRender including trimer structures of prefusion “closed” HIV-1 Env (pdb 3j5m) and SARS-CoV-2 spike (pdb 6vxx) as well as activated, “partially open” HIV-1 Env (pdb 5vn3) and SARS-CoV-2 spike (pdb 6vyb). Viral sequences were downloaded from the Global initiative on sharing all influenza data (GISAID)-EpiCoV and the Los Alamos National Laboratory (LANL) HIV Databases [30,93]. For better comparability, phylogenetics and genetic diversity analyses were performed using HIV-1 and SARS-CoV-2 sequences from one entire year, respectively. For SARS-CoV-2, the first year of the pandemic was studied (mid-December 2019–mid-December 2020), and all full-length sequences with high coverage were downloaded for the studied countries. For HIV-1, we selected a year (January–December) in which a comparable number of HIV-1 unique sequences had been deposited (within 1.5 log difference) relative to the SARS-CoV-2 data set in the same country. Duplicate HIV-1 sequences were removed using the ElimDupes tool from the LANL database. Multiple sequence alignments were performed using Mafft on XSEDE v.7.402 as implemented in the CIPRES Science Gateway v. 3.1 [94]. RAxML maximum likelihood trees were generated using RAxML-HPC v.8 on XSEDE with 1000 bootstrap replicates on CIPRES. Phylogenetic trees were visualized using FigTree v.1.4.3 [95]. Highlighter plots were created using ten study sequences covering all major branches of the phylogenetic trees against SARS-CoV-2 or HIV-1 reference sequences Wuhan_Hu_1 (EPI ISL 402125) or HxB2 (K03455), respectively. Genetic distances were calculated using the ape package (“K80” model without pairwise deletion of sites with missing data) and displayed as heatmaps (upper triangle of all pairwise genetic distances) using the complex heatmap package in program R v.4.0.2 x64 and RStudio v.1.3.959 [96,97]. The longitudinal course of clinical and laboratory parameters was modeled based on available data at the time of the manuscript’s completion (June 2021) as summarized and cited in the review’s respective sections.

## 3. Origins of SARS-CoV-2 and HIV-1

HIV-1 and likely SARS-CoV-2 as well, originated from zoonosis as they are both understood to have originally been transmitted to humans from non-human animals. The World Health Organization (WHO) defines zoonosis as a disease or infection that is naturally transmissible from vertebrate animals to humans [98]. HIV is the result of multiple cross-species transmissions of simian immunodeficiency viruses (SIVs) naturally infecting African primates such as African green monkeys, sooty mangabeys, mandrills, chimpanzees, and others [5]. SIVs are largely non-pathogenic in their natural hosts, while HIV-1 causes the acquired immunodeficiency syndrome (AIDS) in humans. There are two major types of human immunodeficiency virus (HIV), type 1 (HIV-1) and type 2 (HIV-2), differing genetically by nearly 55% [99]. HIV-2 origins were confirmed by demonstrating that humans in West Africa harbored HIV-2 strains that resembled a locally circulating SIV in sooty mangabeys (*Cercocebus atys*) [100]. This virus has remained largely restricted to West Africa since its discovery in 1989 [101]. On the other hand, HIV-1 disseminated within the human population exhibiting high genetic heterogeneity and giving rise to four distinct groups based on multiple cross-species transmission events: M (major), O (outlier), N (non-M/non-O) and P [102,103,104,105,106]. Group M viruses are responsible for the HIV-1 global pandemic, further classified in distinct subtypes (A–D, F–H, and J–L), sub-subtypes (A1–A6, F1, and F2), and circulating and unique recombinant forms [107]. HIV-1 originated from the transmission of a closely related SIV strain from chimpanzees (*Pan troglodytes*) to humans that was first found in 1989 in two captive chimpanzees in Gabon and isolated from one of them. Sera from these animals cross-reacted with all HIV-1 proteins including envelope proteins [101]. The last common ancestor of HIV-1 group M has been dated to approximately 1910 to 1930, indicating that HIV-1 first emerged in West-Central Africa and spread for some 50 to 70 years before it was recognized [101]. Although the early transmission, dissemination, and establishment of the ape precursors of HIV-1 groups M, N, O, and P in human populations remain unclear, it is believed that transmission of SIV into humans occurred through cutaneous or mucous membrane exposure to infected ape blood and/or body fluids exposures common in the context of bush meat hunting, a longstanding common component of household economies throughout Sub-Saharan Africa [11,101,108]. A rapid worldwide spread of HIV-1 has been favored by its enormous genetic variability and rapid evolution, making the virus highly adaptable to new hosts [99]. 

Since HIV-1, the major driver of the HIV pandemic, originated from great apes such as chimpanzee and gorilla, the initial reservoir and the pathology is mostly restricted to/specific for hominids. In contrast, the reservoir of betacoronaviruses such as severe acute respiratory syndrome coronavirus 1 and 2 (SARS-CoV-1 and SARS-CoV-2) and Middle East respiratory syndrome coronavirus (MERS-CoV) is substantially broader and includes bats, cats, dogs, pangolins, minks, ferrets, and even camelids in the case of MERS-CoV [109,110,111,112]. Compared to HIV, coronavirus cross-species transmission events between humans and animals are believed to occur more frequently and back and forth, leading to potentially more diffuse epidemic dynamics that might be harder to control. 

SARS-CoV-2 is estimated to have originated between October and December 2019 from zoonosis, but the exact origin remains under extensive scrutiny [113]. The confirmation of a viral pathogen’s zoonotic origin often relies on the isolation of a virus from a non-human animal that shares >99% whole-genome nucleotide identity. In the case of SARS-CoV-2, there has yet to be the isolation of such a >99% similar virus from a non-human animal. The most closely related virus, RaTG13, was isolated in 2013 from a horseshoe bat (*Rhinolophus affinis*) within the Yunnan province, China, and shares 96% nucleotide identity [114]. While the presence of this sequence in wild bat populations strongly suggests that SARS-CoV-2 originated in bats, the sequence divergence of these two viruses represents approximately 20 years of evolution, suggesting that RaTG13 can only be regarded as an evolutionary precursor and not a direct progenitor [115]. Moreover, RaTG13 is considerably divergent from SARS-CoV-2 within the receptor-binding domain (RBD) of the spike protein, resulting in an approximately 1,000-fold lower affinity of RaTG13 spike to the human ACE2 receptor than SARS-CoV-2 spike [116]. These findings suggest that there is an intermediate host linking the transmission of SARS-CoV-2 from bats to humans.

The pangolin has been implicated as an intermediate host of SARS-CoV-2 due to the isolation of a coronavirus-denoted Pangolin-CoV (or GD/1/2019) from a sick Malayan pangolin [117]. Pangolin-CoV is identical to SARS-CoV-2 in all five critical residues for receptor binding of the RBD but has only 92% whole-genome nucleotide identity [117]. Analysis of horizontal gene transfer and recombination lends support for intragenic recombination of the spike genes between RaTG13 and Pangolin-CoV, which could have given rise to the chimeric SARS-CoV-2 [118]. However, neither RaTG13 nor Pangolin-CoV contain the polybasic furin cleavage site at the S1/S2 junction, which is present within the spike protein of SARS-CoV-2 and greatly contributes to the virus’s tropism and pathogenicity [119]. Moreover, additional analysis conversely suggests that the presence of identical functional sites within the RBD of SARS-CoV-2 and Pangolin-CoV likely arose independently through random mutations and strong natural selection in addition to recombination, refuting the intermediate transmission of pangolins [120]. Overall, the origin of SARS-CoV-2 remains elusive and in order to clearly identify the natural non-human reservoir of SARS-CoV-2 and confirm its zoonosis, extensive sampling of potential host species is necessary. However, this is likely to be a challenging endeavor as SARS-related coronaviruses are known to be widely distributed across Asia [121]. Additionally, the now widespread distribution of SARS-CoV-2 in humans may result in spillover events to non-human animals, which may undermine any surveillance sampling programs.

## 4. Operating Principles of SARS-CoV-2 and HIV-1

The difference between SARS-CoV-2 and HIV-1extends over their genome organization, repertoire/functioning of viral proteins (see Section 4.1), and viral life cycle (see Section 4.2). The latter includes differences in viral entry, specifically their cellular and receptor tropism (see Section 4.2.1), translation and transcription programs, which, in the case of HIV-1, involve RNA reverse transcription and integration of HIV-1 proviral DNA into the host genome (see Section 4.2.3). Both viruses benefit from their customized annexation of the cellular machinery (see Section 4.2.3) and have peculiarities in the proteolytic processing of viral proteins (see Section 4.2.4). 

### 4.1. Viral Composition

**Figure 1:**
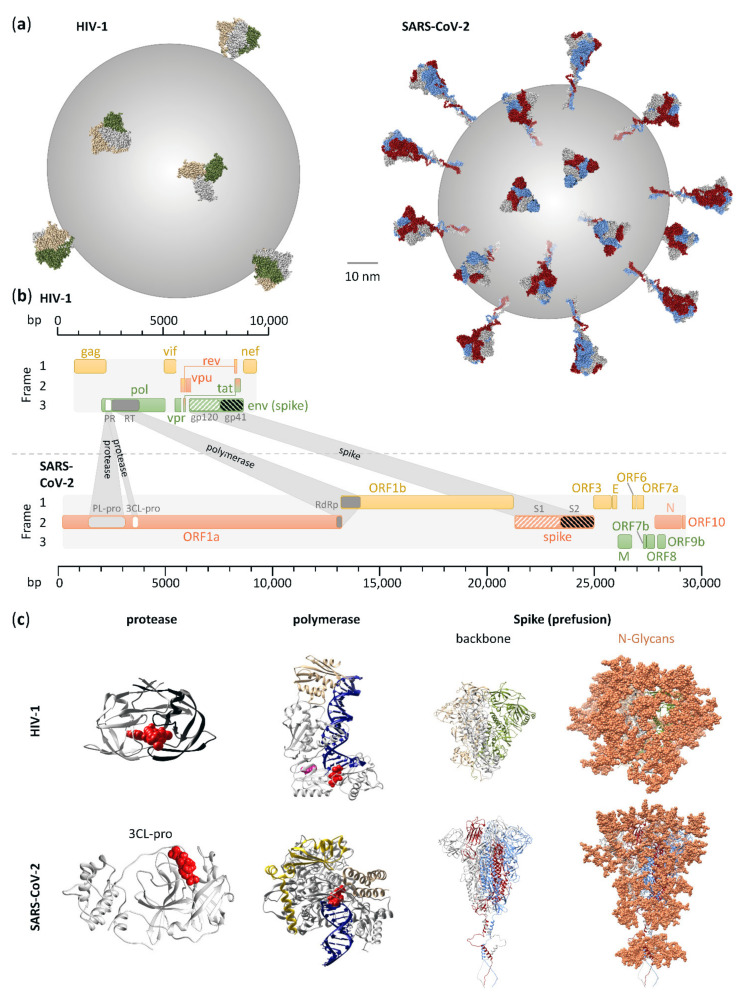
Comparison of HIV-1 and SARS-CoV-2 key viral features. (**a**) HIV-1 and SARS-CoV-2 are enveloped viruses with a diameter of ~100 nm. They are decorated with trimeric spike proteins that mediate viral entry, yet SARS-CoV-2 spikes appear in higher numbers than HIV-1 Env (see Table 1). (**b**) HIV-1 and SARS-CoV-2 possess differently sized, positive-stranded RNA genomes. The HIV-1 genome is ~10 kb in size, whereas the SARS-CoV-2 genome spans almost 30 kb. Genomic regions coding for the key functional or structural proteins protease, polymerase, and spike are highlighted in white, gray, and black and white stripes. (**c**) Three structural and functional proteins highlighted in (**b**) are also shown as 3D structures (ribbon representation), with bound inhibitors shown in red or purple (sphere representation). Protein subunits are colored differentially. Polymerase-bound RNA/DNA is shown in blue. HIV-1 and SARS-CoV-2 spike proteins are shown as amino acid backbone structures (left) and glycoproteins (right) with modeled N-glycans (coral; sphere representation).

Both SARS-CoV-2 and HIV-1 are enveloped viruses with a (+)ssRNA genome and a critical set of structural and functional proteins complemented by a broad spectrum of viral co-factors (Figure 1). Despite a ~3-fold difference in genome size, SARS-CoV-2 and HIV-1 virions are similarly sized (~100 nm in diameter). According to the Baltimore virus classification, SARS-CoV-2 belongs to group IV viruses, characterized by (+)ssRNA, whereas HIV-1 is listed among group VI viruses, i.e., ssRNA-RT viruses with (+)sense RNA and a DNA intermediate in the life cycle [20,21]. More specifically, SARS-CoV-2 belongs to the family of Coronaviridae (realm: Riboviria, order Nidovirales, genus: Betacoronaviruses, subgenus: Sarbecoviruses, species: Severe acute respiratory syndrome-related coronaviruses), which are the largest known RNA viruses with genomes approximately 30 kb in size (Figure 1, Table 1) [122]. Coronaviruses are named after their crown-like appearance in electron microscopic pictures evoked by their large spike glycoproteins extending from the roughly spherical virions. HIV-1 belongs to the family of Retroviridae (realm: Riboviria, order: Ortervirales, genus: Lentiviruses) [123]. Two copies of HIV-1 RNA are enclosed by a characteristic conical capsid of ~2,000 copies of the viral Gag protein (p24). The capsid is surrounded by a mantle of viral matrix proteins (p17) and a lipid bilayer derived from the infected host cell. The latter is spiked with trimeric viral envelope proteins (Env), the sole viral protein on the HIV-1 surface and the mediator of cellular entry. We refer to this protein also as spike to align it with SARS-CoV-2 nomenclature. While the SARS-CoV-2 spike number is moderate compared to other enveloped viruses such as influenza, it is even lower for HIV-1, with 14 or fewer Env spikes incorporated into the viral membrane (Figure 1, Table 1) [124]. The high plasticity of HIV-1 and SARS-CoV-2, as characteristic for Retroviridae and Coronaviridae, and their potential to tolerate sequence and structural changes without critical loss of function has warranted their zoonotic transmission and ongoing evolutionary success [125,126].

The HIV-1 genome codes for nine viral genes (*gag*, *pol*, *env*, *vif*, *vpr*, *vpu*, *tat*, *rev*, and *nef*) encoding 18 proteins (the Gag proteins MA, CA, SP1, NC, SP2, and P6, the Pol proteins RT, RNase H, IN, and PR, the Env proteins gp120 and gp41, the regulatory proteins Tat and Rev, and the accessory proteins Nef, Vpr, Vif, and Vpu). In addition to the structurally and functionally necessary *gag*, *pol*, and *env* genes, the regulatory and accessory *tat*, *rev*, *nef*, *vif*, *vpr*, and *vpu* modulate HIV-1 infection, replication, viral release, and immune recognition [127]. The SARS-CoV-2 genome codes for 14 open-reading frames (ORFs) encoding 29 proteins [128]. The 5′-end of the genome is dominated by the large ORF1a and ORF1b genes, comprising more than 2/3 of the entire ~30 kb genome. They encode polyproteins, which, upon translation, are proteolytically processed into 16 non-structural proteins (NSP1–NSP16) that mostly belong to the replicase–transcriptase complex. At the 3′-end of the genome, 13 ORFs are expressed from subgenomic RNAs: in addition to nine accessory proteins, SARS-CoV-2 encodes four structural proteins, as typical for coronaviruses, i.e., spike, envelope (E), matrix (M), and nucleocapsid (N), the latter complexing the RNA genome in the absence of a surrounding capsid as is the case for HIV-1. In contrast to HIV-1, all the three remaining structural SARS-CoV-2 proteins are incorporated into the viral membrane (spike, E, and M), with spike mediating viral entry. In addition to the canonical ORFs, numerous discontinuous transcription events make the SARS-CoV-2 transcriptome highly complex, including transcripts encoding unknown ORFs with fusions, deletions, and/or frameshifts [129].

SARS-CoV-2 and HIV-1 encode spike glycoproteins, comprised of mainly oligomannose N-glycans in HIV-1 and more balanced complex, oligomannose, and hybrid N-glycans in SARS-CoV-2 [91,92,130]. The higher number of N-glycans on a smaller spike protein renders the HIV-1 glycan shield denser than that of SARS-CoV-2, complicating Ab access to critical entry epitopes with consequences for the development of efficient vaccines (Figure 1, Table 1).

### 4.2. Viral Replication

**Figure 2:**
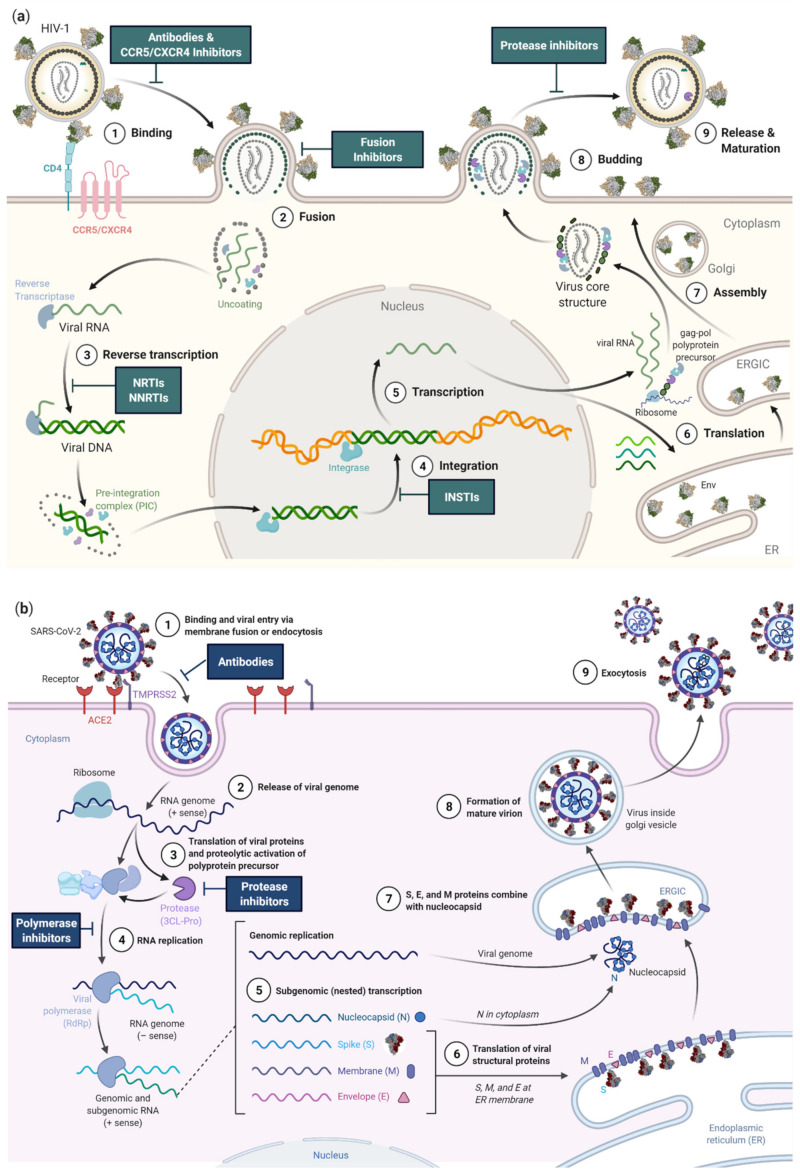
Replication cycles of (**a**) HIV-1 and (**b**) SARS-CoV-2 and major sites of therapeutic intervention. 3CL-pro: 3C-like protease; ACE2: angiotensin-converting enzyme 2; ER: endoplasmic reticulum; ERGIC: endoplasmic-reticulum-Golgi intermediate compartment; INSTI: integrase strand transfer inhibitor; NRTI: nucleoside analog reverse transcriptase inhibitor; NNRTI: non-NRTI; TMPRSS2: Transmembrane protease serine 2.

Although HIV-1 and SARS-CoV-2 are both enveloped viruses with a (+)ssRNA genome, they have evolved different strategies to enter their host cells, replicate, and release their progeny (Figure 2). Despite engaging different entry receptors and target cells, HIV-1 and SARS-CoV-2 follow similar principles of class I glycoprotein-mediated viral fusion and entry (see Section 4.2.1). However, transcription and downstream processes are critically different (see Section 4.2.2). Maybe the most fundamental difference is that the SARS-CoV-2 life cycle occurs entirely in the cytoplasm, whereas the HIV-1 life cycle partially occurs in the nucleus. For this reason, HIV-1 replication takes approximately double the time of SARS-CoV-2 replication. Specifically, HIV-1 reverse transcription was shown to initiate at approximately 3 h post infection (h.p.i.), with double-stranded viral cDNA being detectable 2 h later [44]. In CD4^+^ T cell lines, integration starts 8.5 h.p.i. and all viral transcripts are detectable ~15 h.p.i. The viral gene expression peak is reached between 20 and 23 h.p.i., with ~0.6% of all transcripts in the cell demanded by the virus. The release of viral particles stretches over several hours and is initiated at 18 h.p.i. and can continue to 36 h.p.i. in vitro or 60 h.p.i. in vivo (Figure 2a) [44,45]. In contrast, a SARS-CoV-2 replication cycle takes only ~12 h in A549 cell and in human airway epithelial cell cultures (HAEC), and time-of-addition experiments showed that initial translation and viral replication start simultaneously at between 2 and 3 h.p.i. (Figure 2b) [48]. The following chapter presents the life cycles of both viruses, with a focus on contrasting these two life cycles. 

#### 4.2.1. Viral Entry

As the first step of the viral replication cycle, cellular entry is one of the most critical, as it decides the fate of both the virus and the cell. For this reason, the viral glycoproteins and cellular receptors that facilitate this process are central targets for vaccines, antibody therapies, and small molecular drugs (Figure 2). For enveloped viruses, including HIV-1 and SARS-CoV-2, entry begins with an attachment step to cellular receptors, followed by conformational changes of their receptor-binding glycoproteins, and is completed with the fusion of viral and host membranes.

**Figure 3:**
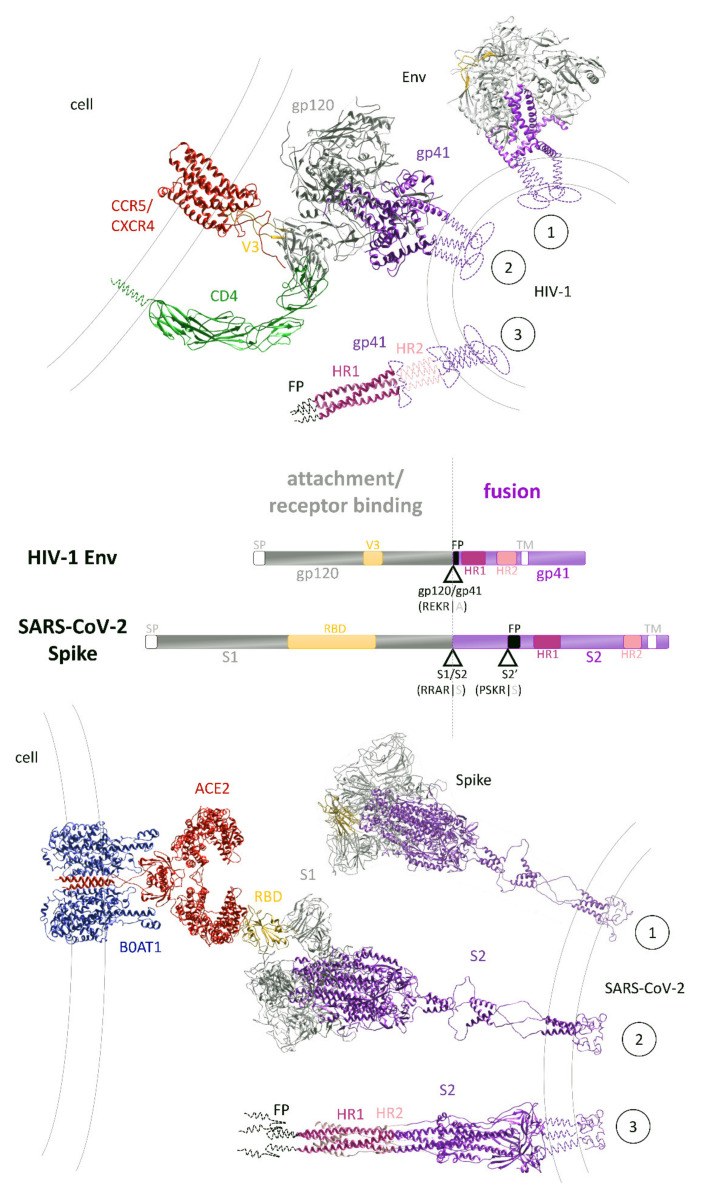
Spike-mediated cellular entry of HIV-1 (**top**) and SARS-CoV-2 (**bottom**). Structural model depicting transition/activation states of viral spike proteins during viral entry. (**1**) Prefusion “closed” state, (**2**) partially “open” state after interaction of spike proteins with cellular receptors, (**3**) fusion intermediates after dissociation of cellular attachment domains gp120 (HIV-1) or S1 (SARS-CoV-2), which exposes fusion peptides for insertion into the target cell membrane. Schematics of HIV-1 Env and SARS-CoV-2 spike coding genomic regions are shown in the middle with domains colored the same way as shown in the structural models. ACE2: angiotensin-converting enzyme 2, B0AT1: sodium-dependent neutral amino acid transporter, RBD: receptor-binding domain, FP: fusion peptide, and HR: heptad repeat.

Attachment of SARS-CoV-2 and HIV-1 is facilitated by the glycoproteins incorporated within their viral envelope membranes. The glycoproteins of both SARS-CoV-2 and HIV-1 are trimeric class I fusion proteins, named spike or Env (also known as gp160), respectively. Both are composed of an N-terminal attachment domain (S1; gp120) mediating receptor binding and a C-terminal fusion domain (S2; gp41) consisting of four critical elements enabling viral fusion, i.e., fusion peptide (FP), heptad repeat 1 and 2 (HR1, HR2), and transmembrane domain (Figure 3) [131,132]. To facilitate efficient attachment, both spike and Env are glycosylated and furin cleaved during viral maturation. Glycosylation aids immune evasion and is considerably greater on HIV-1 Env than on SARS-CoV-2 spike (Figure 1c). Cleavage of both glycoproteins by the host protease furin generates non-covalently bound subunits of their respective N- and C-terminal domains, thereby priming each glycoprotein for engagement with subsequent receptors or host proteases. The cellular receptor for SARS-CoV-2 is the widely expressed angiotensin-converting enzyme 2 (ACE2) [133], and it binds via the RBD of spike (Figure 3) [53,134]. Notably, the additional cell surface receptor neuropilin-1, which is highly expressed in the respiratory and olfactory epithelium, has been shown to bind exclusively to the furin-cleaved spike, potentiating SARS-CoV-2 infectivity in these tissues [135,136]. Alternatively, SARS-CoV-2 spike has also been shown to interact with the host cell receptor CD147 (basigin) to facilitate viral endocytosis [137]. In contrast to SARS-Cov-2, attachment of HIV-1 occurs via binding of gp120 to the cell surface immunoglobin glycoprotein CD4 (Figure 3), which is expressed on subsets of T cells and macrophages [49,138]. Cellular attachment can be initiated or supported by additional cellular membrane proteins such as integrin α4β7 [139,140].

Upon engagement with their host cell receptors, SARS-CoV-2 spike and HIV-1 Env undergo conformational changes to facilitate virus–host membrane fusion. The conformational changes exhibited by both spike and Env enable the extension of their hydrophobic fusion peptides, which are essential for virus–host membrane fusion and subsequent virus entry into the host cytoplasm. The molecular triggers for these conformational changes are different for both viruses. For SARS-CoV-2, extension of the fusion peptide within the S2 domain is triggered through cleavage by host cell proteases at the S2′ site (Figure 3). The canonical entry occurs through membrane fusion directly at the plasma membrane and involves S2′ cleavage by the host protease TMPRSS2 at the cell surface following ACE2 engagement [141,142]. Alternatively, SARS-CoV-2 can enter via endocytosis and membrane-fusion-mediated release from endosomes. In support of this second route of entry, the endosome-localized host protease cathepsin-L has been shown to participate in S2′ cleavage of SARS-CoV-2 spike at the endosomal membrane, likely following CD147-mediated endocytosis [137,142]. These alternate mechanisms of SARS-CoV-2 fusion provide the virus with independent and redundant avenues of entry, which likely contributes to the broad tissue tropism of this virus. In contrast to SARS-CoV-2, the binding alone of HIV-1 gp120 to its cell surface receptor CD4 is sufficient for triggering conformational changes. These conformational changes expose and stabilize the variable loop V3-binding site for co-receptor engagement at the cell surface [138]. CCR5 and CXCR4 can both act as co-receptors for HIV-1. No co-receptors have been identified for SARS-CoV-2. CCR5 or CXCR4 binding to V3 of gp120 induces further conformational changes to gp120, which, after dissociation of gp120, releases the fusion peptide of bound gp41 for insertion into the host cell membrane (Figure 3) [143,144]. Subsequent rearrangement of the heptad repeat regions of gp41 bring the viral and host cell membranes in close proximity for fusion and release of the viral capsid into the host cell cytoplasm [143]. The released viral capsid of HIV-1 continues to encapsulate the viral replication components as the pre-integration complex (PIC), until it delivers the HIV-1 dsDNA to the nucleus. Alternatively, the capsid of SARS-CoV-2 immediately uncoats the viral RNA upon entry into the cytoplasm.

#### 4.2.2. Translation, Transcription, and Reverse Transcription

**Figure 4:**
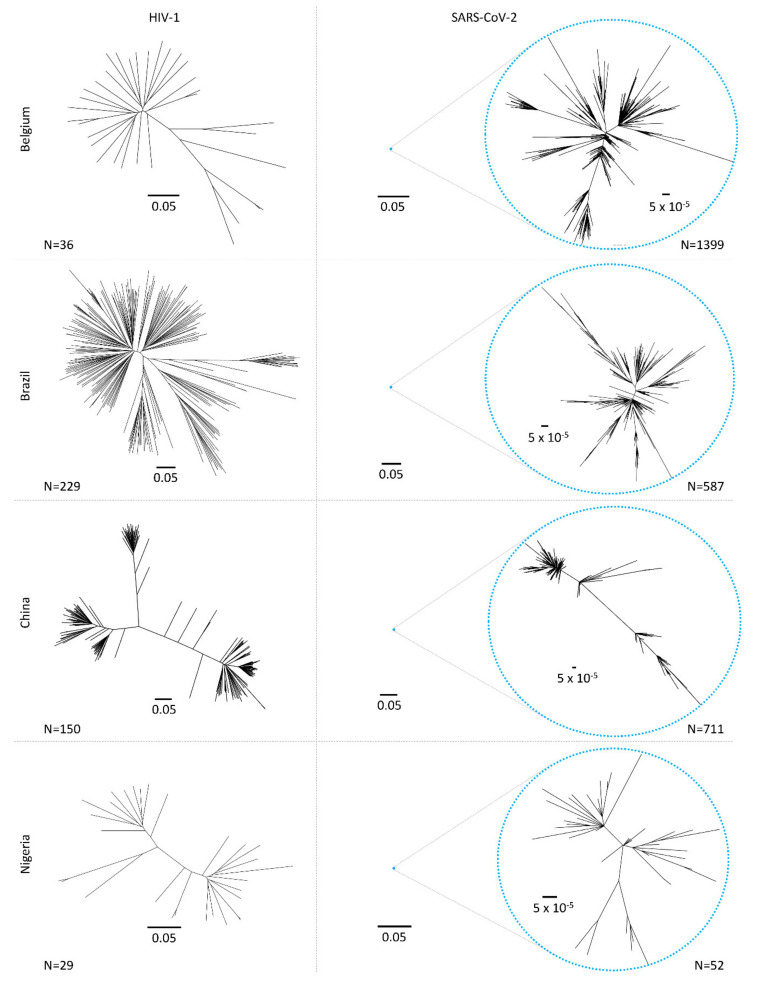
Phylogenetic diversity of HIV-1 and SARS-CoV-2. Maximum likelihood phylogenetic trees (RAxML, 1000 bootstrap replicates) were generated with full length HIV-1 (left) and SARS-CoV-2 (right) genomic sequences from four different countries/continents. For SARS-CoV-2, all available full-length sequences with high coverage were used, deposited to GISAID within one year since initiation of the outbreak in mid-December 2019. Comparably, HIV-1 sequences from one entire year were studied, selected based on comparable case numbers (<1.5 log difference to the respective SARS-CoV-2 data set). Study numbers are indicated in the figure. The collection years of the studied HIV-1 sequences were 2016 (Belgium), 2010 (Brazil), 2007 (China), and 2009 (Nigeria). For SARS-CoV-2, the phylogenetic trees are shown both using a best-fit scale (right) and using the same scale as used for the HIV-1 tree (left; tree condensed to a blue point according to the outline of the tree).

The initial stages of SARS-CoV-2 and HIV-1 replication are remarkably different despite both viruses starting with positive-stranded RNA genomes. Immediately after uncoating in the cytoplasm, the SARS-CoV-2 genome acts as mRNA for the translation of two ORFs (Figure 2b). *ORF1a* and *ORF1b* produce polyproteins named pp1a and pp1ab. *ORF1a* encodes pp1a while the larger pp1ab is the fusion product of *ORF1a* and *ORF1b*, resulting from a -1 ribosome frameshift during translation [47,145]. Following their proteolytic cleavage into 16 NSP subunits, these polyproteins comprise the complete SARS-CoV-2 replicase–transcriptase complex (RTC), responsible for the transcription of the remaining ORFs as well as the full-length gRNA [47]. Conversely, the HIV-1 genome is reverse-transcribed into dsDNA after entry, a step that is catalyzed by the viral RT prebound to the viral genome (Figure 2a). Interestingly, this reverse-transcription process, which lacks a proof-reading step, is much more error-prone than the transcription of the SARS-CoV-2 genome which is supported by a sophisticated proof-reading mechanism [39,146], contributing to the comparatively broad genomic diversity of HIV-1 (Table 1, Figure 4). HIV must transport its nucleic acid to the nucleus via the nuclear pore. Within the nucleus, the PIC is uncoated and the dsDNA complex is integrated into the host genome by the viral enzyme Integrase [147,148,149]. It is only after integration that the HIV-1 genome is transcribed into mRNAs by host enzymes in the nucleus, which are then transported out and translated in the cytoplasm. HIV-1 achieves productive infection by preferential integration of its viral genome in intron regions of highly expressed host genes [147,150]. The chronicity of HIV-1 is caused by latent infection of long-lived memory CD4^+^ T cells and constitutes a major barrier towards a cure of HIV-1 infection [151]. Latent infection is accomplished by integrating HIV-1 into transcriptionally silent regions of the genome of quiescent CD4^+^ T cells [152,153]. 

The transcription of subgenomic (sg) mRNAs and their subsequent translation also differs considerably between these two viruses. The SARS-CoV-2 RTC forms at lipid droplet factories within the cytoplasm [47]. There, the NSP12 RNA-dependent RNA polymerase (RdRp) generates both full-length negative-strand RNA, which acts as the template for replicating SARS-CoV-2 gRNA and shorter sgRNAs of the accessory and structural gene-encoding ORFs [47]. Typical of coronaviruses, transcription regulatory sequences (TRSs) upstream of each of these ORFs prematurely terminate negative-strand RNA transcription in a process referred to as discontinuous transcription. The resulting transcripts are a set of structurally polycistronic nested sgRNAs; however, it is assumed that functionally, these transcripts are monocistronic and that only the 5′-most ORF in each sgRNA is translated [154,155]. Once translated from these sgRNAs, the structural proteins spike, E, M, and N package the gRNA to form infectious virions at the ER-to-golgi intermediate compartment for secretion by exocytosis. In addition to these canonical ORFs encoding structural proteins, recent ribosomal-profiling has identified 23 translationally active unannotated SARS-CoV-2 ORFs with currently unknown function [156]. In contrast to SARS-CoV-2, the transcription of the integrated HIV-1 genome is carried out by cellular polymerases in the nucleus and, through means of alternate splicing, gives rise to over 50 viral RNA transcripts [157]. Governed by the cellular export machinery, only fully spliced RNA transcripts are exported from the nucleus for translation. Hence, the HIV-1 gene products can be separated into early and late based on their necessity for intron retention. The early viral transcripts *rev, tat,* and *nef* are encoded by fully spliced transcripts and consequently, can be immediately exported for translation, while the remaining genes encoded by partially or unspliced transcripts rely on sufficient levels of Rev to accumulate. Rev binds HIV-1 intron-containing transcripts and enables their alternate export to the cytoplasm. The structural Gag and Gag-Pol polyproteins are translated from the unspliced gRNA and consequently are among the last proteins translated. Moreover, Gag is translated from the *gag* gene, while Gag-Pol is a fusion protein generated by a ribosomal frameshift during translation of the *gag* gene to the alternate *pol* reading frame [158]. Once all the HIV-1 structural proteins are translated, virion assembly proceeds at the cell membrane.

Despite their differences in replication strategies outlined above, SARS-CoV-2 and HIV-1 also share some similarities that may pose universal targets for therapeutic intervention. Each of these viruses is encapsulated by a replication complex during their replication within the host cytoplasm. The RNA synthesis of SARS-CoV-2 has been shown to occur in ER-derived double-membrane vesicles [159], while the reverse-transcription of HIV-1 occurs within the viral capsid/PIC [160]. These complexes act to segregate immunogenic viral replication intermediates from cytosolic innate immune sensors [161]. Moreover, the translation of the polyproteins of each virus relies on ribosomal frameshift events. During SARS-CoV-2 replication, this frameshift occurs when translating *ORF1a* and *ORF1b* to give rise to pp1ab, while in HIV-1 replication, a frameshift gives rise to the Gag-Pol polyprotein. Interestingly, the frameshift efficiency in SARS-CoV-2 is 57% ± 12% giving rise to only slightly greater expression of pp1a than pp1ab [156], while during HIV-1 replication, the frameshift efficiency is only 5%, which generates 20 times greater expression of Gag than Gag-Pol [162]. The efficiency of the frameshift during HIV-1 replication is strictly maintained, and any disruption to this rate is detrimental to virus assembly, genome packaging, and maturation [163,164,165,166]. In contrast, the regulation and implications of the frameshift giving rise to SARS-CoV-2 pp1ab are not as well characterized, but likely serve similar regulatory purposes for viral protein expression.

#### 4.2.3. Virus–Host Interaction and Exploitation of the Cellular Machinery

Viral pathogens depend on the cellular machinery to propagate. Thus, it is evident that both HIV-1 and SARS-CoV-2 redirect large parts of the cell-intrinsic biological processes for their opportunistic use involving several thousands of genes and proteins [44,167,168,169,170,171]. While the complex network of interactions between these viruses and their host cells has only been disentangled rudimentarily, the viral strategies of exploiting cellular components and programs appear manifold in both HIV-1 and SARS-CoV-2 infection. In CD4^+^ T cells, the primary target of HIV-1, more than 70% of all expressed genes are modulated in concordance with key steps of HIV-1 viral replication and more than 50% of the longitudinal variability in the host transcriptome can be explained by correlations with main phases of the viral life cycle [44]. This leads to a massive change in cellular physiology with a pronounced early transcriptional shutdown, followed by a progressive, fine-tuned upregulation of parts of the cellular machinery. These changes support viral processing and reproduction and are only partly due to the cell’s triggered defense mechanisms. Single-cell transcriptome studies revealed that HIV-1 targets heterogeneous cells including subpopulations with low expression of interferon-stimulated genes [150]. Proteomic studies confirmed HIV-1 protein-mediated surface downregulation of HIV-1 restriction factors such as SERINC3/5 and interference with CD4^+^ T cell mitogenesis [168]. A study of ~9000 proteins in primary human CD4^+^ T cells across multiple donors revealed 650 HIV-1-dependent changes against the background of T cell activation [167]. Accessory HIV-1 proteins including Vif, Vpr, Vpu, and Nef played a dominant role, accounting for 46% of the HIV-1-specific proteomic changes in primary T cells.

Similar to HIV-1, SARS-CoV-2 profoundly interacts and modifies the cellular physiology both on the transcriptional [169,170,171,172,173] and protein levels [128,170,174,175]. Host responses to SARS-CoV-2 vary substantially depending on viral load, infection stage, disease severity, body tissue, and cell type as well as age and sex of the host [172,176,177]. Coupled to the induction of an antiviral response, the expression of the SARS-CoV-2 receptor and interferon-responsive gene ACE2 is upregulated by the infected host cell in a viral load-dependent manner. In contrast, B cell-specific proteins and neutrophil chemokines are elevated in individuals with lower viral load. Transcriptional levels of the SARS-CoV-2 spike-processing host protease TMPRSS2 depend on infection time point and cell type [171,172]. Time series transcriptome profiling of Calu-3 cells infected in vitro with a clinical SARS-CoV-2 isolate revealed a strong upregulation of TMPRSS2 mRNA within the very first few hours post infection [171], whereas at later time points, the levels revert to baseline or even slightly below [171,172]. Males and older individuals exhibit impaired transcriptional activity affecting trafficking and/or antiviral responses through the reduced function of cytotoxic T cells, B cells, and natural killer cells [172]. Proteomic studies further showed that SARS-CoV-2 reshapes cellular translation, splicing, carbon metabolism, protein homeostasis, and nucleic acid metabolism [174]. In addition to viral proteins, it was shown that SARS-CoV-2 RNA directly and specifically binds and/or modulates a broad network of human proteins in infected human cells [178], and host mitochondria serve as an organelle platform for anti-SARS-CoV-2 immunity [179]. Approximately one-third of the cellular RNA-binding proteins (RBPs) are remodeled upon SARS-CoV-2 infection, and inhibition of these RBPs impairs SARS-CoV-2 infection [180].

#### 4.2.4. Proteolytic Processing of Viral Proteins

A central component of viral replication, including that of SARS-CoV-2 and HIV-1, is the proteolytic processing of viral proteins, which serves to orchestrate genome replication, assembly and maturation. This process involves the cleavage of viral proteins mediated by proteases that are encoded either by the virus or the host. Virally encoded proteases are typically responsible for the autocatalytic excision from polyproteins in which they reside, and for subsequent proteolytic processing of the remaining polyprotein components (i.e., 3CL-pro in pp1a/pp1ab or PR in Gag-Pol). While minimizing coding space within the viral genome, this polyprotein processing also coordinates synchronized translocation of tethered viral proteins to assembly sites within the host cell [181]. In addition to processing by viral proteases, viral proteins, including those of SARS-CoV-2 and HIV-1, are also cleaved by host proteases (i.e., furin), most notably to mediate viral maturation, which primes progeny virions for efficient entry into new cells. The critical functional role of post-translational viral protein cleavage proposes this process as an attractive target for antiviral therapeutic development.

Characteristic for viruses with a positive-sense RNA genome, SARS-CoV-2 and HIV-1 both encode polyproteins that undergo proteolytic processing by virally encoded proteases. However, the replication stage at which the polyprotein proteolytic processing occurs is different between SARS-CoV-2 and HIV-1, reflecting their alternate replication strategies. For SARS-CoV-2, polyprotein processing occurs post viral entry and prior to viral replication. The two SARS-CoV-2 polyproteins pp1a and pp1ab are translated from the incoming positive-sense RNA genome and are proteolytically processed by two cysteine proteases, papain-like protease (PL-pro) and 3-chromtrypsin-like protease (3CL-pro, or main protease; M-pro), which reside within NSP3 and NSP5, respectively, and are released auto-catalytically (Figure 1) [182]. PL-pro catalyzes the cleavage of NSP1-3 and the amino-terminal of NSP4, while 3CL-pro cleaves the carboxyl terminal of NSP4 as well as the remaining NSP5-16 [183]. These cleavage events are essential for the subsequent steps of SARS-CoV-2 replication, and consequently, their inhibition through therapeutic intervention is a topic of ongoing research efforts (Figure 2) [48,184]. In the HIV-1 life cycle, polyprotein processing occurs at later stages, post viral replication and during viral assembly. Indeed, proteolytic cleavage of HIV-1′s integral structural and replicative proteins Gag and Gag-Pol typically occurs during virion assembly and maturation at the cell surface or within the budded virion. Cleavage is performed by the aspartic HIV-1 protease (PR), itself harbored within the packaged Gag-Pol polyprotein and released auto-catalytically (Figure 1) [185]. The PR cleavage of Gag generates the main structural proteins MA, CA and NC, which subsequently rearrange to form the mature, infectious particle [186]. The PR cleavage of Gag-Pol, while also generating the structural proteins, additionally gives rise to the TFP, PR, RT, RNase H and IN proteins required for initial reverse-transcription and integration upon entry into a new cell [186]. Incompletely processed HIV-1 polyproteins fail to covert the assembled virus particle into a mature infectious virion, and HIV-1 PR has consequently been a target of anti-HIV-1 therapies for numerous years (Figure 2) [186,187].

In addition to polyprotein cleavage by viral proteases, the life cycles of both HIV-1 and SARS-CoV-2 include proteolytic processing of their receptor-binding glycoproteins, which are performed by host proteases. These events occur late in the life cycle for both viruses, during a process called viral maturation. Both viruses utilize the host furin-like proteases for cleavage of their glycosylated receptor-binding proteins within the trans-golgi network during virion assembly. The SARS-CoV-2 spike protein possesses a furin cleavage site at the S1/S2 junction [188], which has been demonstrated to be critical for SARS-CoV-2 pathogenicity [119,141,189,190,191]. It enhances the binding affinity of spike to ACE2 by three orders of magnitude [116], but is not entirely essential for SARS-CoV-2 infection, possibly by the secondary spike cleavage event, which can be mediated by proteases other than furin [28,190,191]. The HIV-1 Env polyprotein cleavage by furin produces the receptor-binding gp120 and transmembrane gp41 subunits (Figure 3) [32,192,193,194,195]. In contrast to furin-mediated cleavage of SARS-CoV-2 spike, the cleavage of HIV-1 Env appears essential for HIV-1 entry and infection [196,197,198]. 

Unlike HIV-1 and most other viruses, SARS-CoV-2 possesses an additional cleavage site within its receptor-binding glycoprotein spike, named S2′, which facilitates exposure of its fusion peptide (Figure 3). This second cleavage is mediated by alternate host proteases TMPRSS2 and Cathepsin-L. While initial discrepancies in findings disputed the contribution of either protease, a recent study has determined a spatial delineation underpinning their alternate contributions [142]. It is now understood that both proteases may cleave spike to facilitate fusion; however, TMPRSS2 acts to cleave spike at the cell surface, while Cathepsin-L cleaves spike within the endosome, which likely contributes to the broad tropism displayed by SARS-CoV-2 [142]. Overall, it is apparent that host proteases are employed by both viruses during maturation and in the case of SARS-CoV-2 also during host cell recognition, which primes their receptor-binding glycoproteins for subsequent entry.

## 5. Humoral Immune Responses

Antibody (Ab) immune responses play a central role in protecting the host from viral infections [199]. Ab-mediated protection is primarily attributed to the Ab binding and neutralization capacity (see Section 5.1), complemented by Ab Fc-mediated responses (see Section 5.2). The Abs’ high specificity to defined viral epitopes imposes a strong selection pressure, which may favor the selection of viral escape mutations (see Section 5.3). In all, the relationship between Abs and viruses uniquely shapes their co-evolution in virus-infected individuals and entire populations.

### 5.1. Antibody Binding and Neutralization

**Figure 5:**
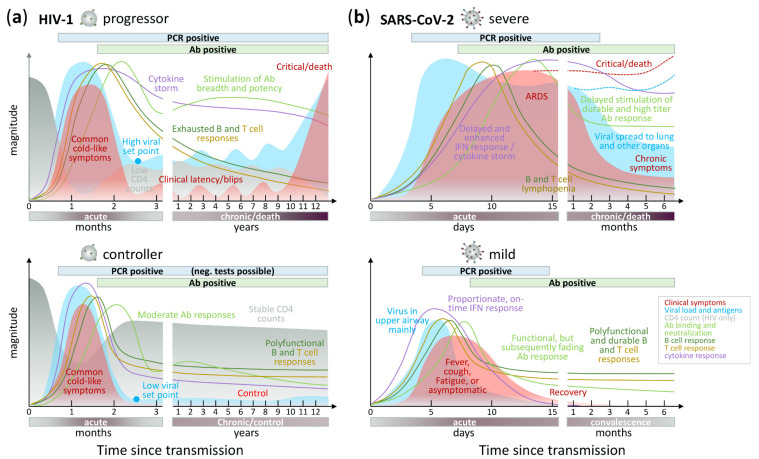
Courses of natural, untreated HIV-1 and SARS-CoV-2 infection. Estimated models of key clinical, viral, and immune parameters and their longitudinal changes in representative courses of HIV-1 (**a**) and SARS-CoV-2 (**b**) infection. Models of more severe/progressive disease courses are shown on top; mild/slow progressive courses are shown at the bottom. Features are color-coded according to the legend and key features directly annotated.

In natural HIV-1 infection, HIV-1-specific Abs are elicited within the first weeks of infection. These include IgM and subsequently class-switched IgG and IgA, which are mainly directed against the immunogenic, highly variable Env gp41 and gp120 regions such as V3 at this initial stage. These early non- or weakly neutralizing Abs are narrow, mostly strain specific, and predisposed to rapid immune evasion [200,201,202]. Broadly neutralizing Abs (bnAbs) occur only in a small percentage of HIV-1-infected individuals (~10%), requiring a few years of continuous antigenic stimulation and maturation, mostly involving high rates of somatic hypermutation [203]. In HIV-1-infected individuals, the development of bnAbs, or high neutralization levels in general, are not associated with better clinical outcome/slow progression, but in turn, correlate with severity of disease and high viral load (Figure 5) [204,205,206]. Knowledge gained from the tedious process of natural bnAb development is currently translated into germline-targeting vaccine strategies with sequential boosting [207]. Similar to SARS-CoV-2, neutralization is considered the lead effector function to protect from HIV-1 infection, since passive administration of bnAbs in animal models of HIV-1 infection can confer protection against viral challenges [199,208,209]. This strategy is currently tested in human clinical trials [210,211]. However, while vaccines can induce sufficiently potent bnAbs against SARS-CoV-2 [212,213,214], and COVID-19 convalescent individuals acquire protective immunity through natural infection [215], it has not been possible to induce broadly protective Abs by HIV-1 vaccines [81] and primary HIV-1 infection does not adequately protect from superinfection [216,217,218]. Ab responses induced in participants of HIV-1 human vaccine trials such as RV144 were mostly non- or weakly neutralizing, waned rapidly, and/or suffered from rapid viral escape (see Section 5.3) [81,86,219,220]. 

In contrast to HIV-1 infection, natural SARS-CoV-2 infection rapidly induces neutralizing Abs (nAbs) targeting primarily the spike RBD and N-terminal-domain (NTD) regions and encompassing a broad range of heavy chain and light chain V genes [221]. Most individuals develop very similar nAb responses with moderate breadth and plasma neutralization activity that require only low rates of somatic hypermutation [222]. Prolonged viral replication in immunocompromised hosts may favor the generation and selection of nAb escape mutants, which in turn drives Ab affinity maturation and eventually enhanced neutralization breadth and potency [223]. In humans, previous SARS-CoV-2 infection and anti-spike Ab seropositivity significantly reduce the risk of SARS-CoV-2 reinfection [215,224,225,226,227,228,229], which is in line with vaccination outcome analyses [230,231,232,233,234,235,236] altogether implying that SARS-CoV-2 binding and/or nAbs exert protective effects. Consequently, neutralization levels have been found highly predictive of immune protection with seven current vaccines and in convalescent cohorts, and provide an evidence-based model of SARS-CoV-2 immune protection [237]. Non-human primate models of SARS-CoV-2 infection corroborated these findings and suggest that nAbs play the leading role in protection from SARS-CoV-2 infection [87,238]. In rhesus macaques, Ab-based protection or their therapeutic potential is dose dependent. Although low Ab titers were sufficient to protect rhesus macaques from SARS-CoV-2 infection or reinfection, higher Ab titers were required to achieve a drop in viral load once infected. Furthermore, cellular immunity contributes to viral control and may compensate waning or insufficient Ab-mediated responses [87,239] (see Section 5.1), e.g., CD8^+^ T cell depletion reduced the protective efficacy of natural immunity against SARS-CoV-2 reinfection in convalescent animals [87]. In addition to nAb responses against the spike NTD and RBD regions, phage display screenings for binding Abs targeting linear epitopes identified common responses in COVID-19 patients against the fusion peptide and the linker region upstream of the HR2, however with variable escape mechanisms [240]. These findings are in line with a recent report stating that the Ab binding response in COVID-19 convalescent individuals converges in >80% to non-RBD epitopes [241]. The early anti-SARS-CoV-2 binding and nAb response is dominated by IgM, which wanes rapidly. IgA and IgG peak subsequently, and IgG-mediated neutralizing responses are most durable and persist over months, mirrored by neutralization half-lives of a few months in serum but up to >8 months in purified IgG samples [242,243,244,245]. The persisting Ab response can be attributed to a maturing humoral immunity driven by a sustained SARS-CoV-2 antigenic stimulation in the gut of COVID-19 patients [246]. Similar to HIV-1, higher anti-SARS-CoV-2 Ab and neutralization levels are preferentially found in severe cases [55,247,248,249,250]. However, COVID-19 survivors exhibit enhanced neutralization potency [247] and a more balanced Ab maturation pathway [251] that might even be critical for survival [252]. More recently, it was shown that severely ill patients do not necessarily mount higher overall humoral responses than mild cases, but are characterized by a delayed kinetic of the anti-spike IgG and nAb response [253]. Neutralization is primarily mediated by receptor-blocking nAbs, and they can either inhibit or enhance syncytia formation [254]. 

Of note, a recent study showed that some Abs against the spike NTD induce the open spike conformation and thus enhance the binding capacity to ACE2 and infectivity of SARS-CoV-2 [255]. Mutational and structural analyses indicated that all infectivity-enhancing Abs target a common site on the NTD and shar a divalent binding mode. Abs specific for the infectivity-enhancing site on the NTD were detected at high levels in severe patients. The identified mechanism of antibody-dependent enhancement (ADE) of viral infection is Fc receptor-independent. It differs from the Fc receptor-dependent ADE identified with other viruses in having a lower impact on infection, but affecting a broader range of cells including ones that do not express Fc receptors [255,256,257]. Excess amounts of nAbs appear to suppress ADE in most cases of SARS-CoV-2 infection or vaccination; however, the precise functional consequences of infectivity-enhancing Abs on SARS-CoV-2 pathogenicity and vaccines, and their differential impact on variants remain elusive.

In addition to SARS-CoV-2 spike, the N and ORF 3b and 8 proteins are highly immunogenic with implications as serological markers [258]. Qualitative differences in early Ab profiles point to elevated Ab responses to the N protein in deceased individuals [259]. Ab immune responses have mainly been studied in the blood, whereas little data exist about the responses at the local sites of infection such as the respiratory system. Of interest, the mucosal immune system comprises the largest part of the immune system. On-site production of secretory IgA (sIgA) by far exceeds all other immunoglobulin isotypes, which renders the mucosa, as site of viral entry, prepared for the initial wave of adaptive defense [260]. Consequently, anti-SARS-CoV-2 IgG and IgM levels, which mainly transudate from the blood into the mucosa, correlate well between both compartments. In contrast, IgA was found to be more abundant in the mucosa, particularly early during disease, which supports the hypothesis that SARS-CoV-2 infection triggers local sIgA production [261,262].

Combining immunological and epidemiological analyses on seasonal coronaviruses has shown that infection-blocking immunity wanes rapidly, but disease-reducing immunity is long-lived, which suggests a model of SARS-CoV-2 transitioning within years to endemicity with mitigated pathogenicity [263].

### 5.2. Antibody Fc-Mediated Functions

Antibody Fc-mediated functions complement Ab neutralization functions and provide a link between Ab- and cell-based immunity (e.g., NK cells and phagocytes) or soluble effectors (e.g., complement) [264,265]. As such, Fc-mediated Ab functions can act hand in hand with neutralization or as an additional line of defense before or after neutralization.

In HIV-1 infection, Fc-mediated effector functions have been studied in detail in recent years, particularly antibody-dependent cellular cytotoxicity (ADCC) and antibody-dependent cellular phagocytosis (ADCP) [266,267]. Using a quantitative approach in HIV-1-infected humanized mice and Simian-HIV (SHIV)-infected rhesus macaques, 25–45% of the total antiviral activity of anti-HIV-1 mAbs was attributed to Fc-mediated effector functions [268]. In support of that, mAbs with non-functional Fc-receptors had dramatically decreased capacity to protect animal models from SHIV infection [269]. Since the isolated depletion of complement binding had no impact on the protective activity, Fc-mediated cellular responses appear to play the dominant role. Indeed, Fc-mediated cellular responses such as ADCC and ADCP have been associated with protection from HIV-1 disease progression and protection from (S)HIV infection in animal models or in a human vaccine trial [81,266,270,271]. For example, ADCC responses in the presence of low plasma IgA/IgG ratios correlated with protection from infection in a large human vaccine trial with partial efficacy (RV144) [86,220,272,273,274], yet a complete mechanistic explanation remains elusive [81,266]. Furthermore, Fcγ phenotyping in vaccinees of the same trial revealed that distinct single-nucleotide polymorphisms (SNP) in the FCγR2C gene conferred 91% vaccine efficacy against HIV-1-carrying immunodominant epitopes in Env that experienced vaccine selection pressure. In contrast, individuals with a different SNP exhibited only 15% vaccine efficacy [275]. 

Many studies have shown a tight linkage between Ab Fc-mediated effector functions and neutralization in HIV-1 infections [269,276,277,278,279,280]. A recent study showed that the neutralization activity of an anti-HIV-1 mAb was potentiated >5000-fold in vitro when expressing the IgG high-affinity Fc receptor FCγRI compared to the same mAb without [278]. Moreover, the antisera from animals immunized with the respective mAb epitope-based vaccine neutralized diverse HIV-1 clades, including more resistant tier-2 viruses, in an FCγRI-dependent manner [278]. Nonetheless, the mutual impact between Fc-mediated functions and neutralization can vary considerably as it was shown, for example, that Fc-mediated activity was partially redundant for a very potent bnAb [281], and differences in antibody binding affinity for HIV-1 and SIV Env uncoupled mAb-mediated ADCC from neutralization [282]. Fc-mediated functions are influenced by the antigenicity and conformation of the infecting strain/molecular clone, Ab binding levels, Ab specificity, Ab orientation on the bound antigen, gp120 shedding, capacity to form multivalent antigen–Ab complexes, degree of internalization of antigen–Ab complexes, and killer cell receptor ligand expression (e.g., NKG2D) [266,280,283,284,285,286].

In SARS-CoV-2 infection, data on the impact of Fc-mediated effector functions are still unfolding, but similar to HIV-1, these effector functions appear to be critical [239,287,288]. Studies in non-human primates demonstrated that Fc-mediated functions correlated with protection from SARS-CoV-2 infection [289]. This was confirmed by studies in mice and hamsters, where nAbs provided better protection when coupled with Fc-receptor functionality [287,290,291]. Fc-effector functions are elicited in symptomatic and asymptomatic COVID-19 individuals, but they are elevated in severe cases [288]. COVID-19 non-survivors had a higher incidence of compromised Fc-receptor binding and effector functions, implying a crucial role for Ab Fc-effector functions in limiting severe disease and reducing patient mortality [251]. An in vitro model of ADCC, using full-length spike proteins expressed on the surface of a target cell line, and PBMCs from healthy individuals serving as effector cells, provided additional mechanistic insights. In this model, the ADCC activity of convalescent plasma decreased only modestly compared to the more pronounced decrease in neutralization activity. Substantial ADCC activity was maintained in 85% of donors’ plasma up to eight months post symptom onset and strongly correlated with plasma IgG responses [242]. Notably, three weeks post vaccination with an mRNA vaccine, a time point at which vaccine efficacy is estimated to be >90%, nAb responses are still mostly absent, but anti-SARS-CoV-2 ADCC responses well developed [239]. This implies a possible role for Fc-mediated effector functions and other cellular responses in vaccine-mediated protective effects. The collected data so far suggest a vital role for Fc-mediated effector functions in sustained protection from reinfection and vaccine-induced protection.

### 5.3. Antibody Escape and Mutant Variants

**Figure 6:**
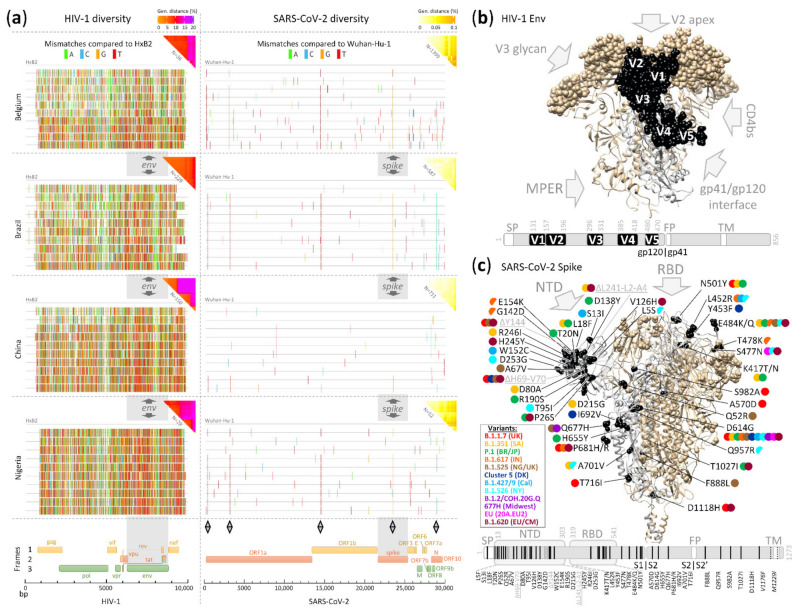
(**a**) Mutational landscape of HIV-1 and SARS-CoV-2. Highlighter plots indicating mutations/mismatches of HIV-1 and SARS-CoV-2 genomes from four studied countries compared to the references HxB2 (HIV-1, left) and Wuhan-Hu-1 (SARS-CoV-2, right). Base pair mutations are shown as colored tics according to the color code on top. Genome maps are shown at the bottom. Gray diamonds indicate recurrent SARS-CoV-2 mutations. Analyses of ten representative sequences are shown that covered all major branches of the phylogenetic trees in Figure 4. The pairwise genetic distances of the entire set of study sequences per country are summarized in triangle heatmaps (upper right corner of each panel) with colored ranges from white to yellow, orange, red, pink, and purple according to genetic distances from low to high (color code indicated on top). The envelope region (*env*) of HIV-1 and the spike region (*spike*) of SARS-CoV-2 are indicated by gray bars and arrows. (**b**) Variable domains in HIV-1 Env (all in gp120) are highlighted in the structural Env trimer model (#6wpu) and the gene map shown below. Variable domains are shown in sphere representation in the structure, colored in black, and labeled in one monomer. In the gene map, the variable domains are shown in black and labeled. The Env epitope regions of five major bnAb classes are indicated by arrows and labeled. (**c**) Amino acid mutations in globally emerging SARS-CoV-2 variants are shown in a SARS-CoV-2 spike structure (S.pdb) [89] and a gene map. The mutations are shown in sphere representation and highlighted in one monomer in the structure. Amino acid replacements are displayed and labeled in black and deletions in gray. The mutations in emerging variants of concern are indicated by circles colored according to the legend to the left; half-circles indicate that mutations occur in only a fraction of variant sequences. The main sites of vulnerability to nAbs are indicated by gray arrows (RBD and NTD). The two most C-terminal mutations are only shown in the gene map, indicated with a dotted line and labeled in italic, i.e., V1176F and M1229I, occurring in P.1 and cluster 5 variants, respectively. Cleavage sites and important amino acid positions, including those of all HIV-1 variable domains and SARS-CoV-2 NTD and RBD domains, are indicated. CD4bs: CD4-binding site; FP: fusion peptide; MPER: membrane-proximal external region; NTD: N-terminal domain; RBD: receptor-binding domain; SP: signal peptide; TM: transmembrane domain.

HIV-1 and SARS-CoV-2 Ab escape is based on similar principles of immune pressure exerted by Abs on their targeted viral epitopes [292]. However, the strength and timing of the driving immune forces, and the capacities to evade these forces are very different in both viruses. Important discriminative factors are the acute nature of SARS-CoV-2 infection, resulting in a small temporal window of active replication and adaptation, combined with a low mutation rate due to the proof-reading mechanism of the SARS-CoV-2 polymerase complex [146]. This contrasts with the chronic nature of HIV-1 infection that allows for a lifelong ongoing viral replication and adaptation with a high mutation rate in the absence of proof-reading by the HIV-1 polymerase complex. Consequently, HIV-1 immune escape is a constant factor in almost every HIV-1-infected individual, whereas SARS-CoV-2 immune escape is rare and seems to occur preferably in immunocompromised individuals with prolonged viral replication and fostered by treatment with mAbs or convalescent plasma (blood plasma from a donor who has recovered from COVID-19) (Figure 6) [293,294,295,296,297].

#### 5.3.1. HIV-1’s Rapid and Continuous Escape

The Ab response early after HIV-1 infection predominantly targets hypervariable regions such as the protruding V1–V5 loops and parts of gp41 of the autologous strain. Immune escape to the early non- or weakly neutralizing Abs occurs promptly and extensively without considerable loss of viral fitness (Figure 6) [200,201,202,298]. HIV-1 Ab escape is a continuous process and occurs through different mutational pathways involving amino acid replacements, insertions, increasing variable loop lengths, deletions, charge changes, conformational blocking, and remodeling/adding shielding glycans. These modulations are largely driven by nAb pressure or to functionally adapt/optimize cellular receptor usage [299,300,301,302,303,304,305,306]. The mutual pressure exerted by nAbs on the virus and by the mutated/escaped viruses on the immune response leads to an arms race of Ab–virus co-evolution, which allows the development of bnAbs in a small percentage of HIV-1-infected individuals [307,308,309]. Anti-HIV-1 bnAbs require lengthy and complex maturation pathways to develop unusual features such as long heavy chain complementarity determining 3 regions, high levels of somatic hypermutation, extensive insertion–deletion events (indels), and auto- or poly-reactivity. These bnAbs principally target semi-conserved regions or conserved glycan sites on HIV-1 Env. Key regions are the membrane-proximal external region (MPER) of gp41, the CD4-binding site (CD4bs), the gp120/gp41 interface, the V3 glycan region, and the apical V2 glycan region (Figure 6), complemented by a few additional, recently discovered bnAb epitopes involving the fusion peptide, silent face, V3 crown, and the V2V5 corridor [309,310,311,312,313,314,315,316]. Consequently, natural HIV-1 infection is characterized by an enormous viral diversification, which can generate close to 5% HIV-1 genetic diversity in singly-infected individuals [41,42,271] with substantial compartmentalization of HIV-1 evolutionary events across anatomical sites [317,318].

#### 5.3.2. SARS-CoV-2 Mutates on a Low but Constant Level, Yielding Mutant Variants over Time

In contrast to HIV-1, intra-patient evolution in SARS-CoV-2 is restricted to mostly <0.05% genetic distance. Differences in variant distribution between upper and lower respiratory tract have been observed; however, the number of minority variants per patient usually remains <30% based on only a few variants (Table 1) [43,319,320,321,322,323,324,325]. A higher intra-patient evolution of SARS-CoV-2 yielding several tens of variants appears to be associated with prolonged replication, as frequently found in more severe cases, older individuals, and particularly immunocompromised patients [43,223,293,294,295,296,297,326,327]. Nonetheless, in most SARS-CoV-2 infections, there is one dominant variant throughout the course of the disease and upon onward transmission [324]. Animal experiments on viral evolution and transmission suggest that within-host SARS-CoV-2 variation is predominantly influenced by genetic drift and purifying selection [328]. Due to the low diversity evolving per patient during the comparably short course of infection, the majority of studies focus on population-wide mutation and evolution analyses (Figure 4 and Figure 6). 

SARS-CoV-2 variants known to date acquired mutations in different protein-encoding regions along the full SARS-CoV-2 genome; however, mutations in spike were detected most abundantly, suggesting selective pressure on spike in particular (Table 2, Figure 6). During the first phase of the SARS-CoV-2 pandemic (early and mid-2020), spike mutations remained rare, except for D614G, which subsequently became dominant. *In vitro* studies with pseudoparticles carrying spike showed that D614G does not seem to confer significant Ab immune escape, but renders spike proteins more stable, increases spike density on the virion surface, and increases production of pseudovirions and thus their infectivity. *In vivo* studies with authentic SARS-CoV-2 further showed that D614G enhances replication and transmission. [329,330,331,332,333,334,335,336]. During the second year of the pandemic (end of 2020 and 2021), new SARS-CoV-2 variants emerged, subsequently replacing old variants [337]. Some of these second-year spike mutations seem to confer partial neutralization resistance, as measured in in vitro assays with convalescent or vaccine sera [338,339,340,341,342,343]. Available potent vaccines at optimal dosing can induce multiple times higher nAb titers than convalescent sera [237,339,344,345] and might thus have a broader window to compensate for partial Ab escape. The enrichment of spike mutations (Table 2, Figure 6) suggests that Ab-mediated immunity, either obtained by SARS-CoV-2 infection or by vaccination, may have exerted critical selective pressure [85]. In the future, it seems possible that selective pressure brought upon by adaptive immunity to SARS-CoV-2 in increasing parts of the general population could further promote selection of variants with a distinct Ab-escape phenotype.

Spike mutations of emerging mutant variants mainly accumulate in three regions: (1) RBD, (2) NTD, and (3) around the S1/S2 cleavage site (Figure 6). RBD and NTD play essential roles as target sites for nAbs, directed in >90% of cases against RBD, complemented by lower percentage nAb activity against NTD [221,249,346,347]. Consequently, these sites are also prominent sites for nAb escape mutations. The S1/S2 cleavage site is functionally important, and mutations at the S1/S2 interface, as primarily shown for D614G in vitro and in vivo, can modify viral entry, transmission, and replication [119,333]. Four mutant variants that spread supra-regionally were classified variants of concerns (VOCs). These VOCs include lineages identified in the UK (B.1.1.7, Alpha) [348], in South Africa (B.1.351, Beta) [349], in Brazil/Japan (P.1, Gamma) [350], and more recently in India (B.1.617.2, Delta) [351,352]. B.1.1.7, B.1.351, and P.1 share the N501Y mutation in RBD in addition to the D614G mutation around the S1/S2 cleavage site (Table 2, Figure 6). Epidemiological studies suggest that B.1.1.7 is associated with higher viral loads in patients [353,354], a >40% increased epidemiological growth [355,356,357], a longer duration of acute infection [358], less effective clearance by innate and adaptive immune responses [359], and increased severity/death rate, particularly in patients of higher age and with comorbidities [360,361,362,363]. The B.1.1.7 variant acquired an enhanced affinity to ACE2 [364] while maintaining sensitivity to nAb responses though with 2–9 times reduced nAb titers in vaccinees and convalescent individuals [364,365,366,367]. Spike mutations present in B.1.1.7 frequently confer resistance to NTD-directed nAbs, whereas neutralization by RBD-specific nAbs remains largely unaffected [368]. Recently, B.1.1.7 variants were detected in the UK and the USA that additionally carry the E484K mutation (VOC-21FEB-02) and render these variants more resistant to monoclonal and polyclonal nAb responses [369,370].

N501Y appears to play an important role in the evolutionary success of VOCs by increasing spike affinity for ACE2 [371] as well as viral infectivity and virulence, as shown in a mouse model of SARS-CoV-2 infection [372]. Molecular dynamics simulations with B.1.351 and P.1 variants indicate that E484K confers a higher RBD affinity to ACE2 [373]. Furthermore, neutralization assays using viruses pseudotyped with variant or single-mutant spike proteins as well as virus selection experiments under nAb selection pressure indicated that E484K is a primary escape and resistance mutation. E484K was responsible for near complete resistance against multiple NTD and RBD mAbs and >10 and >30 times lower nAb titers in vaccinees and convalescent individuals, respectively. N501Y or K417N are additional mutations, all in RBD, conferring reduced nAb activity in SARS-CoV-2 vaccine and convalescent sera [338,339,342,343,343,374,375,376,377,378,379,380,381]. These results could be recapitulated in experiments with SARS-CoV-2 isolates [382,383,384,385], whereas another study with recombinant SARS-CoV-2 carrying the specific point mutations reported smaller effects on nAb titers against infectious SARS-CoV-2 [386]. E484K, N501Y, and K417N escape is alarming because of the dominance of class I and II nAbs among the vaccine- or natural infection-induced polyclonal nAb responses targeting the receptor-binding ridge of RBD including sites 417, 484, and 501 [378,387,388,389]. More recently, there was a massive outbreak of B.1.617 in India, a lineage characterized by the combination of L452R and E484Q spike mutations in sublineages B.1.617.1 and B.1.617.3 or L452R and T478K in B.1.617.2 [351,352,390]. Epidemiologic data and neutralization assays with pseudotyped viruses and isolates suggest a higher transmissibility and partly decreased sensitivity to nAbs and vaccines, which is primarily due to the effects of the RBD mutations E484Q and L452R [391,392,393,394,395]. B.1.617 variants are currently spreading globally, and are rapidly out-competing other variants [396].

To date (mid-2021), B.1.351 and P.1 are the most resistant variants to anti-SARS-CoV-2 nAb responses, and notably, they also acquired the ability to use ACE from other species, thus extending the host range to mice and minks [397,398]. As shown for P.1 variants in Brazil, VOCs may also facilitate coinfections with different SARS-CoV-2 lineages [399]. While animal experiments and human vaccine data suggest that vaccines still provide sufficient protection of disease after challenge with B.1.1.7 or B.1.351 variants [364,400,401,402], a clinical trial in South Africa showed that vaccine efficacy of an approved adenovirus vaccine dropped from >70% to ~10% in preventing mild or moderate disease [403]. Preliminary data indicate that natural infection with B.1.351 as well as immunizations with mutant antigens induce cross-neutralizing Ab responses including potent neutralization of mutant viruses such as B.1.351, indicating that targeted booster vaccines might be broadly effective [404,405,406]. 

In addition to the four VOCs mentioned above, several more variants have emerged with transient peaks or an ongoing rise in regional or supra-regional prevalence [31,407,408,409]. In late 2020 and early 2021, multiple emerging variants were reported in South Africa in addition to VOC B.1.351, and a novel variant was identified in Nigeria carrying the P681H mutation, known from B.1.1.7 [410,411]. In Europe, Cluster 5 variants emerged following an outbreak of mink infections with supposed human spill-over in Denmark [412]. Cluster 5 variants have a prominent Δ69–70 deletion in spike NTD that became dominant later in the B.1.1.7 lineage. Δ69–70 is a prime example of deletions that occur in the SARS-CoV-2 genome, which may drive adaptive evolution and facilitate antibody escape through compensatory mechansisms [413]. Deletions occur during genome replication due to strand slippage and cannot be corrected by the polymerase’s proof-reading mechanism, and thus represent an evolutionary mechanism of generating altered viral genomes despite the low inherent base substitution rate of SARS-CoV-2 [414]. The cluster 5 variant’s Y453F mutation in RBD can reduce titers of therapeutic nAbs in in vitro pseudovirus assays [415,416], but has limited impact on the polyclonal nAb response in vaccines [343]. Furthermore, variants 20A.EU1 (B.1.177) and 20A.EU2 (B.1.160) increased in dominance, the latter one carrying the S477N mutation in RBD that might have resulted from selective pressure exerted by the host nAb response and possibly increases RBD’s affinity for ACE2 [371,417]. In the US, two Californian lineages (B.1.427 and B.1.429) gained dominance in the second year of the pandemic, characterized, again, by recurring spike mutations [418]. Epidemiological data suggest a ~20% increased transmissibility, and in vivo and in vitro data suggest increased shedding, increased infectivity, and a modest decrease in neutralizing titers from convalescent patients and vaccine recipients, mediated by L452R [419,420]. In the Midwest of the USA, variants with diverse mutations at amino acid position N677 increased in frequency, which, due to N677’s proximity to the polybasic S1/S2 cleavage site, potentially affect functional features including viral entry, transmission, and/or spread [421,422]. In New York, B.1.526 variants were detected frequently carrying either the S477N or E484K mutation (Table 2, Figure 6) [423,424]. Since E484K is known to impair nAb efficacy as described for B.1.351 and P.1, these variants pose a possible bias to vaccines. 

More recently, lineage B.1.525 was detected in the UK and in Nigeria and rapidly spread over more than 20 countries in the world. It is characterized by the combination of key mutations of different variants, namely E484K, ΔH69–V70, N439K, and Y453F, and inclusion of a new F888L mutation in the spike S2 domain [425]. B.1.620, first identified in Lithuania, Europe, is spreading in different European countries and traces back to its probable origin Cameroon and surrounding West-Central African countries [426]. Notably, it contains a combination of spike mutations and deletions from different VOCs, including E484K, S477N, P681H, ΔH69–V70, and ΔY144D. In Uganda, the A23.1 variant was detected that lacks D614G, but acquired P681R and the RBD mutation V367F [427]. On the Philippines, variant P.3 was identified, which stems from the same lineage B.1.1.28 as P.1 and harbors N501Y, E484K, and P681H mutations [428]. In Peru and Chile, a new sublineage within B.1.1.1 is expanding, designated C.37, with the novel spike deletion Δ246–252 [429]. Additionally, Vietnam has experienced a spike in COVID-19 cases and detected a hybrid variant with features of both the B.1.1.7 (UK) and B.1.617.2 (Indian) variants, which likely rendered the virus more transmissible [430].

There is growing evidence that similar combinations of mutations evolve across the globe with overlap to mutations determined in in vitro selection experiments. They include mutations at spike position 484, 501, and 614 as well as deletions in NTD (Table 2, Figure 6). It implies that SARS-CoV-2 variants are converging based on common purifying selection processes. The associated appearance of mutations, e.g., in the VOCs at spike positions 417, 484, 501, and 681 indicate the importance of synergisms and compensatory mutations to fine-tune neutralization escape, infectivity, and replicative fitness [125,380]. Close monitoring of variants will remain crucial to identify emerging nAb escape variants and discern phylodynamic spread based on immune pressure from founder effects and sampling bias. Surveillance of mutations, recombination, and novel genome architectures within *spike* as well as the full genome is required to reassess the efficacy of vaccines for the mitigation of SARS-CoV-2 spread [431,432,433,434,435].

## 6. Cellular Responses

The cellular immune responses triggered by either SARS-CoV-2 or HIV-1 represent a double-edged sword in determining disease outcomes. While early cellular immune responses primarily have a protective role, deregulated and exacerbated inflammatory responses can prevent viral clearance and worsen disease outcomes [58,436]. Furthermore, in the context of COVID-19, the transition between innate and adaptive immune responses plays a key role in viral clearance and lung pathology. The accumulation of pro-inflammatory cytokines, lymphopenia, deviant T cell responses, and antiviral immunity mediated by the generation of nAbs in COVID-19 patients suggest that SARS-CoV-2 might induce an immune-related disease [437,438,439]. Indeed, SARS-CoV-2-infected individuals exhibit cellular and humoral immune responses, which characterize mild from severe cases. Individuals with asymptomatic or mild disease mount a predominant cellular but low to no humoral immune response, but patients with severe disease mount a potent humoral but moderate cellular immune response [440]. Similarly, the involvement of host cellular immunity in HIV-1 infection is central to the progression of AIDS [58,441]. Due to the infection and subsequent depletion of CD4^+^ T cells and macrophages, HIV-1 exerts direct and indirect effects on the cellular immune pool.

### 6.1. T Cells

A functional T cell immune response is typically highly effective at eliminating viral infections and/or suppressing viral replication. In turn, dysfunctional responses are associated with severe/progressive disease, as is the case for SARS-CoV-2 and HIV-1 infections. Furthermore, the lymphocyte count is an important clinical parameter of both SARS-CoV-2 and HIV-1 infections as lymphopenia is a common feature in severe/progressive infections of each virus. However, the processes leading to lymphopenia during the two infections are very different. Mild courses of SARS-CoV-2 infection in individuals with agammaglobulinemia, characterized by a lack of B cells, showed that Abs are not the only important variable to clear or control infection [442]. Compared to humoral immunity, T cell responses target a broader range of viral antigens beyond spike/Env, including strong responses against HIV-1 Gag, Pol, and Nef, or SARS-CoV-2 M, N, and ORF3a [443,444,445]. They further possess a larger breadth, e.g., recognizing at least 30–40 CD4^+^ T cell epitopes in each donor with minimal overlap with Ab epitopes [446]. 

The coordination between the innate and the adaptive immune responses in the early stages of SARS-CoV-2 infection is essential to control viral spread [447]. Indeed, delayed or insufficient activation of T cell responses may lead to severe lung damage or systemic inflammation, whereas early induction of functional SARS-CoV-2-specific T cells is associated with rapid viral clearance and mild disease in COVID-19 patients [448,449,450]. In agreement with analyses in the blood, functional and consistent CD8^+^ resident-memory (TRM) and CD4^+^ T-helper-17 (TH17) cells in bronchoalveolar lavages were associated with beneficial outcomes [451,452]. Both CD4^+^ and CD8^+^ T cell responses to SARS-CoV-2 have been determined as important parameters associated with control of SARS-CoV-2 infection, yet CD4^+^ T cell responses appear even more prominent than CD8^+^ T cell responses [221,443,444,453]. Although T cell responses can be slightly elevated in severe COVID-19 cases [444], the activation of helper T (Th) and cytotoxic T cells (CTLs) by SARS-CoV-2 may be either beneficial or harmful depending on whether potent or exhausted immune responses are mounted [436].

The levels of T cells and their elicited responses fluctuate during infection of both SARS-CoV-2 and HIV-1, and often distinguish severe/progressive from mild/controlled infections. In patients with severe or fatal COVID-19, the total lymphocyte count is reduced compared with non-severe patients [454,455], which could be a predictive marker for disease severity [454,456]. Moreover, functional T cells were elevated in patients with mild COVID-19 expressing higher levels of cytotoxic molecules such as granzyme A and Fas ligand that were beneficial in eliminating virus-infected cells. These molecules were reduced in severe disease cases due to the compacted CTL proportion [457]. Thus, SARS-CoV-2-specific T cell numbers correlated with less severe COVID-19 in a prospective study [458]. SARS-CoV-2-specific CD4^+^ and CD8^+^ T cells are found in peripheral blood from COVID-19 patients in the first two weeks after symptom onset, and the majority of these CD4^+^ T cells exhibit a central memory phenotype with a dominant production of Th1 cytokines, while CD8^+^ T cells have a more effector phenotype with high levels of perforin expression [459]. Although lymphopenia preferentially affects CD8^+^ T cells [460,461], both CD4^+^ and CD8^+^ T cell levels were reduced in number and frequency but exhibited increased activation in COVID-19 patients [69,461,462]. This activation state can mount potent immune responses or generate functional exhaustion. At the convalescent stage, SARS-CoV-2-specific T cells generate broad and polyfunctional responses and they even occur after mild infection or in seronegative individuals [444,463]. 

In the early stages of HIV-1 infection, the virus is able to undermine the T cell immune response by infecting CD4^+^ T cells, thus reducing their numbers and impairing their function, which ultimately contributes to viral immune escape [58]. Nevertheless, HIV-1-specific CTLs are crucial to lower the massive viral burden in the early days and weeks of infection to reach the (predictive) viral set point [445]. Indeed, the T cell response is understood to be responsible for the successful and persistent control of HIV-1 viral titers in long-term nonprogressors (LNTPs) or elite controllers (ECs) [58,464,465,466]. CD4^+^ T cell responses including regulatory T cells (Tregs) and Tfh cells are known to play an important role in HIV-1 pathogenesis [467] but also vaccine outcome [220,468]. In a large HIV-1 vaccine trial (RV144), functional CD4^+^ T cell responses have been associated with reduced risk of infection. Specifically, levels of Env-specific poly-functional CD4^+^ effector memory T cells capable of secreting multiple cytokines including CD40L, IL-2, IL-4, IFN-γ, and TNF-α were associated with higher vaccine efficacy [220,468].

The Time Course of T Cell Responses in SARS-CoV-2 and HIV-1 Infection

In HIV-1 infection, the T cell response initially rises following the increase in viral load and peaks as the viral load begins to drop (Figure 5a) [469,470,471,472]. In progressive HIV-1 infections, the viral load may again increase while the T cell response inversely decreases, which is partly due to T cell exhaustion in these cases (Figure 5a) [471,473]. Additionally, the effective T cell control of HIV-1 is also heavily reliant on HLA presentation of peptides to CD8^+^ T cells. The HLA alleles HLA-B27 and HLA-B57, as well as a polymorphism in HLA-C, have been identified in LTNP/EC individuals and are suggested to stimulate more effective CD8^+^ immune responses than those observed in cases of progressive HIV-1 infection [474]. HIV-1-infected LTNP/EC individuals also maintain poly-functional CD8^+^ responses with enhanced degranulation, cytokine, and chemokine production, contributing to a controlled infection in these individuals (Figure 5a) [475,476,477]. In terms of the CD4^+^ T cells, as primary targets of HIV-1, their levels are low during peak viremia, typically 21–28 days p.i., but return to normal levels in the following weeks during the establishment of the viral set point [64]. Of note, the CD4^+^ T cell levels have been observed to return to normal within the blood but not within the gastrointestinal tract, which represents a major site of T cell depletion due to HIV-1-induced apoptosis, causing an overall reduction in CD4^+^ T cells during chronic HIV-1 infection (Figure 5a) [478,479,480]. 

A longitudinal study of CD8^+^ T cells during SARS-CoV-2 infections revealed an exhausted phenotype of CD8^+^ T cells, based on the presence of exhaustion markers including PD-1, CTLA-4, and TIGIT, which, together with reduced poly-functionality according to markers such as IFN-γ, TNF-α, and IL-2, predicted disease severity [481]. However, other studies showed that CD8^+^ T cells are not exhausted but remain functional [482,483] and a significant proportion of SARS-CoV-2-reactive T cells with “exhausted” phenotype are also found in patients with mild COVID-19 [484]. Interestingly, these cells exhibited lesser cytotoxic and inflammatory features and could maintain their exhausted state even after viral clearance. In contrast, SARS-CoV-2-reactive CD8^+^ T cells from patients with severe disease displayed multiple features that support the generation of robust CD8^+^ T cell memory responses with pro-survival properties and a lack of restraining exhaustion features [484]. To what degree SARS-CoV-2 causes T cell exhaustion and/or impaired T cell memory remains to be determined. Nevertheless, the high durability of T cell responses suggests that T cells are a critical component among the correlates of protection after infection or vaccination [485]. The potency and breadth of anti-SARS-CoV-2 T cell responses, which are readily induced post natural infection and vaccination, may drive humoral responses and/or compensate the lack of sufficient nAb responses in the early weeks post vaccination when neutralization is still weak [239]. Of note, the vast majority of SARS-CoV-2 T cell epitopes are not affected by the mutations found in circulating mutant variants [486]. In summary, depending on the magnitude and functionality of the highly specific T cell responses, they can either drive protective or pathogenic immune responses in SARS-CoV-2 infection.

### 6.2. B Cells

B cell responses in SARS-CoV-2 infection occur concomitantly with T follicular helper (Tfh) responses, starting approximately one week after symptom onset [487]. Antigen-specific CD4^+^ T cells are important for eliciting potent B cell responses that result in Ab affinity maturation, and spike-specific T cell levels correlate with serum IgG and IgA titers [443]. Memory B cells (Bmem), circulating Tfh cells, and spike-specific Abs are positively associated with plasma neutralizing activity in patients who have recovered from COVID-19 [488]. The SARS-CoV-2-specific B cell repertoire consists of transcriptionally distinct B cell populations with two main clusters of potent nAb-producing cells, resembling memory and activated B cells [489].

In addition to antibody production, B cells establish immunological memory, the basis for durable protective immunity after infection or vaccination. Duration of immunological memory after SARS-CoV-2 infection and COVID-19 is still unclear, but antigen-specific memory T and B cells are detectable in convalescence [488,490] and each component of immune memory appears to exhibit distinct kinetics [244]. There is growing evidence that SARS-CoV-2 infection generates antigen-driven long-lasting B cell memory that persists and matures for several months after SARS-CoV-2 infection and may provide long-term protection against systemic disease upon reinfection [491,492]. A recent study confirmed that SARS-CoV-2 infection induces long-lived bone marrow plasma cells [493]. Thus, waning nAb titers in plasma several months post infection may be compensated by the persistent Bmem repertoire that remains at constant levels or can even increase [494]. Further studies are required to elucidate the contribution and protective capacity of Bmem cells in SARS-CoV-2 infection.

In terms of HIV-1 infection, the B cell response is highly dysregulated, resulting in patient hypergammaglobulinemia and defective humoral responses [495,496]. The dysregulation of the B cell responses, specifically polyclonal B cell activation, was one of the first immunological abnormalities identified in HIV-1-infected individuals [497]. This HIV-1-induced dysregulation was later determined to be driven by active HIV-1 replication and coincides with the increase in viral titers (Figure 5a). The normalization of B cell responses in HIV-1 viremic individuals following antiretroviral treatment (ART) suggested the HIV-1-specific B cell responses to be driven by HIV-1 replication [498,499]. Indeed, the abnormal B cell responses, including non-specific polyclonal Ab responses and hypergammaglobulinemia, are now considered hallmarks of active HIV-1 replication [500,501,502]. The ability of HIV-1 to dysregulate the B cell response is proposed to be a consequence of the virus’s depletion of CD4^+^ cells [496]. In particular, the Tfh cells, which are crucial for B cell maturation and the generation of the Ab response, were found to be functionally impaired in HIV-1-infected individuals and unable to aid B cell function adequately [503]. Moreover, early HIV-1 replication has been shown to cause mass apoptosis of primary infected cells and bystander cells through the production of apoptotic microparticles and the secretion/shedding of apoptosis-inducing HIV-1 viral proteins [504]. This considerable apoptotic activity combined with the high levels of HIV-1 replication within the lymph nodes likely contributes to the damage or loss of approximately 50% of germinal centers observed within the first 80 days of HIV-1 infection [505,506]. As major sites of B cell function and the generation of HIV-1 nAbs [507], the HIV-1-induced destruction of germinal centers considerably impairs the infected individual’s ability to rapidly generate high-affinity HIV-1 Abs [64]. In addition to the early impairment of B cell maturation and Ab generation, HIV-1 infection has also been demonstrated to compromise the proliferation and cytokine secretion of memory/activated B cells [508,509]. Altogether, it is apparent HIV-1 imposes considerable restrictions on B cell functions early during infection, impairing the host’s ability to mount an effective humoral immune response. Conversely, LTNP/EC HIV-1-infected individuals maintain an effective B cell response, notably that of Tfh cell function [510], which contributes to their control of the infection and offers important considerations for effective vaccine design.

### 6.3. Monocytes/Macrophages

Pathogenic changes in the monocytic compartment regarding phenotypes and function and an increase in immature neutrophils are central hallmarks of COVID-19 [511]. Macrophage activity drives both inflammation and much of the pathology in COVID-19 patients [512,513]. These cells act as sentinels to limit early viral replication by initiating an IFN-I response and an inflammatory response to recruit additional immune cells [514]. However, when macrophages are highly activated, these cells can produce large amounts of cytokines (cytokine storm) that generate systemic hyperinflammation [515]. Megakaryocytes and monocyte subsets are critical peripheral sources of cytokine storms, which involves interactions of hyper-inflammatory cell subtypes in lung and peripheral blood [169]. The activation of macrophages is called macrophage activation syndrome (MAS) [515], and MAS-like severe inflammation and fibrinolysis are involved in COVID-19-associated pneumonia [516,517]. In lungs of COVID-19 patients, moderate levels of macrophages can be found in alveolar exudates while infiltrated T cells and monocytes are found in the interstitial compartment [518], and infiltrated macrophages in the alveolar lamina [519].

In the lungs, different macrophage populations exist that diverge in their gene expression profile and tissue localization [520]. In COVID-19 patients, anti-inflammatory monocyte-derived (FCN1^high^) macrophages were identified in patients with mild disease, while resident pro-fibrotic (SPP1^high^) and inflammatory alveolar macrophages (FAPB4^+^) dominated in patients with severe disease [457]. Additionally, increased cellular interactions were found in patients with critical COVID-19, and they were consistent with a higher activation status of non-resident macrophages, monocyte-derived macrophages, and CTL. In particular, non-resident macrophages showed a highly inflammatory profile characterized by significantly higher expression levels of chemokine encoding genes (CCL2, CCL3, CCL20, and CXCL1) and pro-inflammatory cytokines (IL8, IL18, and TNF) in patients with critical disease [521]. 

ACE2 expression on macrophages is limited to CD169^+^ macrophages in lymph nodes and spleen, which renders these macrophages susceptible to SARS-CoV-2 infection [512]. Despite the lack of ACE2 expression, other studies suggested that SARS-CoV-2 can also infect alveolar macrophages, establishing a positive feedback with T cells that drive persistent alveolar inflammation [522]. Infection of macrophages by other coronaviruses is known to induce altered functional states, e.g., impaired MHC II presentation by MERS-CoV [523,524]. Although the mechanism is not fully understood, MHC II downregulation was also demonstrated in monocytes and B cells from COVID-19 patients and its expression was partially restored by using an inhibitor targeting IL-6, a major driver of COVID-19 pathology [525]. Altogether, current knowledge suggests that macrophage polarization and the composition of macrophage subpopulations play key roles in COVID-19 severity.

Similar to SARS-CoV-2 infection, the monocytes and subsequently macrophages drive much of the inflammation associated with HIV-1 infection [526], but in this case, also constitute important reservoirs and disseminators of HIV-1 [527,528,529]. Circulating monocytes are some of the first cells to respond to HIV-1 infection and are key mediators of host antiviral defenses and inflammation [528]. Importantly, CD16^+^ and intermediate monocytes, which express the HIV-1 co-receptor CCR5, can be infected by HIV-1 [530]. Furthermore, HIV-1 is able to replicate within these monocytes, generating reservoirs of the replication-competent latent provirus [531,532]. This combined with their ability to participate in cell-to-cell transmission of HIV-1 [533], instigates CD16^+^ monocytes as key disseminators of HIV-1. Moreover, their ability to cross the blood–brain barrier further instigates these monocytes as the primary source of HIV-1 infection in the brain and subsequently key perpetrators of the development of HIV-1-associated neurocognitive disorders [534]. Importantly, HIV-1 has been demonstrated to persist in monocyte-derived tissue-resident macrophage during antiretroviral therapy [535,536], which emphasizes the necessity of including these cells in latency-reversing anti-HIV-1 therapies.

## 7. Cytokines and Innate Immune Response

The host innate immune response is initiated through the recognition of viral components or products of the viral replication cycle by pattern recognition receptors (PRRs). For viruses with an RNA genome such as HIV-1 and SARS-CoV-2, this occurs through three main classes of PRRs: Toll-like receptors, RIG-I-like receptors (RLRs), and NOD-like receptors (NLRs) [537]. However, unlike SARS-CoV-2, HIV-1 may also be detected by multiple DNA receptors, including cGAS, IFI6, and DDX41, due to its unique DNA replication intermediate [65]. Moreover, host adaptor proteins PQBP1 and NONO have been demonstrated to selectively aid in the detection of these transient retroviral DNA replication intermediates [538,539]. 

Central to the innate immune response is the production of multiple cytokines with various immunomodulatory effects required to control viral infection. For both SARS-CoV-2 and HIV-1, the magnitude and duration of the innate immune response are key determinants of either severe/progressive or mild/controlled infection. During mild/controlled SARS-CoV-2 and HIV-1 infections, the innate immune response, which is primarily determined by cytokine production, is initially robust, controls the viral levels, and then quickly subsides (Figure 5, purple and blue curves). Conversely, in severe/progressive infections of these viruses, the cytokine production is typically delayed but then exhibits an enhanced magnitude and prolonged duration, compared to mild/controlled cases (Figure 5, purple lines). This phenomenon is commonly referred to as ‘cytokine storm’. The prolonged innate immune response coincides with sustained viral levels (Figure 5, blue curves). Consequently, SARS-CoV-2 and HIV-1 severe/progressive cases fail to control viral replication and exhibit an exuberant cytokine production, which hence develop aggravated clinical symptoms (Figure 5, red curves).

The production of pro- and anti-inflammatory circulating cytokines drives much of the disease pathophysiology associated with SARS-CoV-2 and HIV-1 infections and offers insight into the stage of infection and disease outcome. COVID-19 pathogenesis can be divided into two phases: the early phase, where the viral infection affects the upper respiratory tract (1-14 days of initial encounter), and the late phase, when the virus spreads to the lungs and induces profound hypoxemia and respiratory failure accompanied by other complications such as viral sepsis, different organ failures, and death. Although most SARS-CoV-2 infections are asymptomatic or mildly symptomatic [540], in a minority of cases, the virus can cause severe pneumonia and in some of them develop into acute respiratory distress syndrome (ARDS) and systemic disease [541] (Figure 5b). The outcome of SARS-CoV-2 infections depends on both the viral load and the immune responses [542]. Likewise, HIV-1 infection can be separated into two main stages; the early acute infection spanning the first few months from the initial transmission and the subsequent chronic infection, which is currently incurable and consequently lifelong. Both stages have distinct viral features and clinical symptoms. The early stages of HIV-1 infection from the time of transmission, can be sequentially divided into the eclipse period where viral RNA remains undetectable in plasma, peak viremia, and the viral set point which exhibits stable viremia [64]. During this time, patients generally only experience common cold-like symptoms. Conversely, during the chronic stage of HIV-1 infection, where virus levels fluctuate due to reanimated latent reservoirs and immune escape mutants, untreated individuals exhibit substantial immune suppression contributing to HIV-1-associated diseases and the development of AIDS (Figure 5a).

Among the heavily distorted innate immune response typical of COVID-19, the late-wave inflammatory response in particular is linked to COVID-19 disease severity [483,543,544,545]. The major differences in immune phenotype between moderate and severe cases are apparent after day 10 of infection. These can be grouped into two main clusters: (1) patients with decreased expression of pro-inflammatory cytokines and enrichment of tissue repair genes for whom the disease trajectory remains moderate, leading to eventual recovery; and (2) patients with higher and sustained pro-inflammatory cytokine levels who exhibit a worse disease trajectory, which can lead to death [69]. Signature cytokines in severe COVID-19 include enhanced expression of IL-6, IL-1β, IL-1α, IL-2, IL-7, IL-10, TNFα, MIP1α, MCP1, G-CSF, IFNγ [69,73,521,546,547,548]. In children, the increased inflammatory markers include IL-6, IL-1, and C-reactive protein along with procalcitonin in serum [549]. Among all the above-mentioned cytokines, IL-6 has been reported as a major driver of COVID-19 pathophysiology. Consistent with this, the highest IL-6 levels were observed in patients requiring intensive care. In these patients, there is a continuous IL-6 increase over time and levels are elevated in non-survivors [550,551]. In addition, secretory cells from COVID-19 patients show higher expression of the chemokine-ligand encoding genes CXCL1, CXCL2, CXCL3, CXCL6, CXCL8, CXCL16, and CXCL17, likely promoting the recruitment of neutrophils, T cells, and mast cells and aggravating the inflammatory response [521,552]. The cytokine and chemokine receptor expression increases markedly in patients with critical disease compared to moderate disease, suggesting augmented recruitment of immune cells to inflammation sites [521]. Similarly, HIV-1 infection displays distinct cytokine profiles in the plasma, which increase in magnitude relative to increases in viral titers. Following the detection of HIV-1, there is typically a rapid and transient production of IL-15, CXCL10, and type-I IFNs as well as a rapid but sustained production of IL-18, TNF, IFN-gamma, and IL-22 [553]. IL-10 is also transiently produced following HIV-1 detection but is slightly delayed compared to the other cytokines [553]. Moreover, the early production of the chemokines CCL3 and CCL4 has been shown to exacerbate HIV-1 infection by recruiting target CD4^+^ T cells to the infection foci [554]. Progressive HIV-1 infection is typified by elevated production of the IL-6 cytokine as well as D-dimer, C-reactive protein, and CD14, which together are markers for increased risk of HIV-1-associated mortality [555,556,557,558,559,560,561,562].

Nasopharyngeal SARS-CoV-2 viral load generally correlates with plasma levels of interferons and elevated cytokines. Moreover, viral load correlated significantly with IFNα, IFNγ, and TRAIL levels. In patients who ultimately died of COVID-19, many chemokines responsible for monocyte and T cell recruitment and survival such as CCL1, CCL2, M-CSF, IL-2, Il-16, and CCL21 were elevated [69], suggesting pathological roles associated with host defense factors.

### Interferon Response

Pattern recognition followed by specific signal transduction events ultimately results in the production of interferons (IFNs), which are potent immune cytokines that upregulate IFN-stimulated genes (ISGs) and other cytokines that are essential for an effective antiviral response [563,564]. Among the IFNs, type I IFNs constitute the universal language, as they are produced by, and their receptors are found on all nucleated cells. However, responses are fine-tuned to perform a range of different activities dependent on the type I IFN subtype (message) and the responding cell type (receiver) [565]. As in many immune processes, both timing and magnitude of IFN responses are key to balance optimal antiviral efficiency and side effects of inflammation.

Coronaviruses and HIV, like many other viruses, have evolved antagonistic mechanisms to the host’s antiviral response and both use different mechanisms to manipulate the IFN response: (1) avoidance, where the virus protects itself from recognition by PRRs; (2) suppression of IFN induction, where the virus inhibits the transcription of interferons [566]; and (3) suppression of IFN signaling, where viral proteins inhibit IFN alpha receptor (IFNAR) signaling [567].

In the airways, type I IFNs play important roles in protecting from the spread of respiratory viruses and are critical in initiating inflammatory responses. Although SARS-CoV-2 is sensitive to IFN-I [537] and all IFN types can inhibit its replication in a dose-dependent manner [568], recent studies have reported a delayed induction of the IFN-I response during SARS-CoV-2 infection [569,570], partly driven by SARS-CoV-2-promoted autophagy [571]. This delayed IFN induction is a prominent feature of COVID-19 and distinguishes SARS-CoV-2 infection from other viral infections such as SARS-CoV-1 and influenza A virus (IAV) [68,443]. Although the precise mechanisms used by SARS-CoV-2 to evade the innate immune response remain poorly understood, a number of SARS-CoV-2 proteins have been recently reported to antagonize the IFN response [572]. NSP6 binds TBK1 to suppress IRF3 phosphorylation, NSP13 binds and blocks TBK1 phosphorylation, and ORF6 binds KPNA2 to inhibit IRF3 nuclear translocation [573]. Other viral proteins antagonize IFN-I signaling by blocking STAT1/STAT2 phosphorylation or nuclear translocation [68,439,572,573]. Interestingly, IFN signaling during SARS-CoV-2 infection appears to be modulated not only by viral proteins but also by certain host factors. Inborn errors of TLR3- and IRF7-dependent type I IFN immunity were described in some patients with critical disease [574]. Furthermore, it was shown that some patients with life-threatening COVID-19 had neutralizing IgG auto-antibodies against IFN-ω, IFN-α, or both. These antibodies prevent the corresponding type I IFNs from blocking SARS-CoV-2 infection in vitro and were preferentially found in patients with severe COVID-19 [575,576,577,578]. Altogether, these data strongly support the notion that dysregulated type I IFN immunity underlies life-threatening COVID-19 pneumonia.

SARS-CoV-2 primarily targets airway epithelial cells, alveolar epithelial cells, vascular endothelial cells, and macrophages in the lung, all of which express ACE2, the predominant receptor for SARS-CoV-2 entry into host cells [33,579]. Both SARS-CoV-2 infection and subsequent release of inflammatory cytokines such as TNFα and IL-β can enhance ACE2 shedding, which is attributable to the induced tissue damage [580]. Furthermore, ACE2 itself is an ISG, suggesting that SARS-CoV-2 may exploit IFN-driven ACE2 up-regulation to enhance infection [50]. Data showed that epithelial cells of the upper respiratory tract in patients with COVID-19 exhibit an average three-fold increase in ACE2 expression (mRNA) that correlates with IFN signals by immune cells. These include preferential expression of IFN-γ by CTLs and of genes encoding its receptors (IFN-γR1 and IFN-γR2) by secretory and ciliated cells, supporting the notion that ACE2 up-regulation is at least partially due to IFN-γ signaling by immune cells [521]. In addition, one of the central transcription factors of the IFN response, STAT1, was among the top predictors for ACE2 expression [521]. However, it was also observed that the benefit of IFN-induced ACE2 upregulation does not outweigh the IFN-induced antiviral activity to suppress viral replication once established [568], suggesting that the virus’ ability to delay the IFN response early in infection is a crucial factor in disease progression. Interestingly, other factors such as tobacco smoke [581] and age [582] also regulate ACE2 mRNA levels and the IFN response. It was observed that older and/or smoking patients lacking IFN-I exhibit higher viral loads and require more aggressive medical intervention accompanied by a long time of stay in the intensive care unit [583]. Both enhanced ACE2 levels and reduced IFN-I production and ISG induction might be related to the higher susceptibility of the elderly population to COVID-19. 

The type-I IFN response elicited during HIV-1 infection also requires a specific balance to control the viral titers and not cause detrimental inflammatory immunopathology [584]. HIV-1 is sensitive to type-I IFN, and consequently, the type-I IFN response offers control of HIV-1 titers early during infection [585,586,587]. However, misappropriated type-I IFN responses may lead to IFN-I desensitization, increased HIV-1 infection, and accelerated disease [588]. Indeed, comparative transcriptomics studies identified lower expression of ISGs in HIV-1-positive individuals who exhibited control of the infection and onset of disease compared to those in which disease progressed rapidly [589,590]. While type-I IFNs contribute to reducing HIV-1 viral load early in infection, the therapeutic use of type-I IFN has shown little to no benefits in HIV-1-infected patients with progressed infection and likely contributes to enhanced immunological disease [586,591,592]. In line with these findings, the therapeutic antagonism of type-I IFN receptor signaling during chronic HIV-1 infection has been demonstrated to control HIV-1 reservoirs, reverse inflammation-associated diseases, and rescue host anti-HIV-1 T cell immunity [593,594]. The advent of single genome amplification studies has highlighted the importance of robust initial control of HIV-1, as approximately 80% of HIV-1 infections were found to be established by a single founder virus [595,596,597]. In the instance of a misappropriated type-I IFN response, this single founder virus would likely represent an immune escape mutant, which likely contributes to subsequent IFN resistance in latent infection. In summary, it is apparent that the type-I IFN response is critical during initial SARS-CoV-2 and HIV-1 infections, but the timing, duration, and magnitude of the response must be regulated to appropriately prevent the progression of the infection and subsequent associated disease.

## 8. SARS-CoV-2 and HIV-1 Co-Infection and Mutual Impact

Data on the mutual impact of SARS-CoV-2 and HIV-1 co-infections are still unfolding. Early during the COVID-19 pandemic, mixed and partly contrasting results from clinical studies were reported, mainly based on differences in cohort composition, limited co-infection cases per study, and confounding factors, including differences in applied or absent ART [598,599,600]. While an immunocompromised status has been considered a general risk factor for COVID-19, it remained elusive whether the resulting decreased detrimental inflammatory response might even confer beneficial effects and mitigate clinical symptoms [601,602,603,604,605]. 

Most data showed that HIV-1-infected subjects receiving ART have a comparable risk of COVID-19 infection as healthy individuals, and HIV pre-exposure prophylaxis (PREP) users are not at risk of poorer COVID-19 disease outcomes than the general population when infected [606,607]. However, some studies showed an underrepresentation of severe COVID-19 cases among the HIV-1 patient population, implying that certain kinds of ART might lower the risk of severe COVID-19 outcomes [608]. Based on such findings and due to the similarities of potential HIV-1 and SARS-CoV-2 drug targets, especially the proteases, a repurposing of anti-HIV-1 drugs was initiated early in the pandemic. However, antiretroviral drugs exhibited only moderate effects against SARS-CoV-2 in vitro and had no efficacy in clinical trials [1,609,610,611,612,613]. 

Data on patients with uncontrolled HIV-1 infection, i.e., with detectable HIV-1 RNA and poor immunological status, are scarce but small studies indicated no pronounced impact of an ongoing HIV-1 infection on COVID-19 clinical outcome [614]. Although HIV-1 altered the response of CD4^+^ T, CD8^+^ T, and natural killer cell subsets, COVID-19 disease in people living with HIV (PWH) remained comparable to HIV-negative participants [615]. Of note, however, a study reported that, although PWH were not overrepresented among COVID-19 cases, these individuals had a higher rate of COVID-19-related complications, presumably due to a higher prevalence of underlying factors associated with more severe COVID-19 outcomes [616]. In contrast to some of the earlier studies, recent data showed that indeed there might be substantial morbidity and mortality from COVID-19 among PWH, even in the setting of HIV-1 suppression [617,618,619]. Factors associated with risk for severe COVID-19 in HIV-1-infected patients were low CD4^+^ T cell counts and discontinuation of ART. HIV-1-mediated CD4^+^ T cell depletion has been associated with impaired T cell and humoral immune responses to SARS-CoV-2 [620]. The only factor associated with mortality was a low CD4^+^ T cell set point, but not HIV-1 viral load or type of ART [604,621,622]. SARS-CoV-2-specific IgG concentrations and pseudovirus nAb titers, but not avidity, were lower among PWH compared with HIV-negative controls [623]. Moreover, black PWH had a substantially increased risk of severe disease, thus rendering African regions with a high prevalence of HIV-1 infection more vulnerable to COVID-19 and requiring closer surveillance [617]. The prolonged replication, enhanced evolutionary rates, and severe clinical relapse of SARS-CoV-2 in immunocompromised patients highlights the risk carried by individuals with a suppressed immune system, such as those with an active HIV-1 infection [293,294,295,296]. Of note, a vaccine efficacy study against the South African variant B.1.351 indicated that the Novavax vaccine is less effective in HIV-1-infected (49% efficacy) compared to HIV-1-uninfected individuals (60% efficacy) [624], corroborating an impact of HIV-1 infection on anti-SARS-CoV-2 immune responses and/or COVID-19 outcome. 

While most studies have focused on the impact of HIV-1 infection on COVID-19, there are only limited data on the interference of COVID-19 on HIV-1. A modeling study estimated that the most significant risk of the COVID-19 pandemic on PWH comes from interrupted ART in resource-limited countries [625]. COVID-19 cases were reported where SARS-CoV-2 infection increased HIV-1 viral load and thus might foster HIV-1 viral rebound [626].

In summary, accumulating data suggest an underlying HIV-1 infection slightly increases the risk of severe COVID-19, particularly in patients that are sub-optimally treated and immunocompromised [627]. Studies with higher case numbers with full adjustment of confounders need to confirm these preliminary findings. 

## 9. Conclusions

The raging AIDS and COVID-19 pandemics taught us important lessons on viral pathogenesis and made the causing agents HIV-1 and SARS-CoV-2 some of the world’s best-studied viruses. Both viruses are of comparable structure with key components of enveloped viruses with positive-strand RNA genomes, but they differ profoundly in their basic functioning. SARS-CoV-2 is responsible for an acute, in most cases, self-limiting disease, which may exhibit respiratory symptoms at the initial stage with possible systemic dissemination if chronic disease stages are entered. Conversely, the integration of HIV-1 as a provirus into the host genome and its capacity to reside there for years underline the primary chronic nature of HIV-1 infection after a usually mild acute infection. The high error rate during reverse transcription makes HIV-1 a master of immune escape, with HIV-1 Ab immune evasion progressing rapidly and comprehensively in almost every patient. Furthermore, the massive glycan shield covering HIV-1 Env proteins and the depletion of critical immune cells during pathogenesis render HIV-1 a highly challenging virus to develop both curative approaches and vaccines against. In contrast, SARS-CoV-2’s mutational activity is comparably silent, which facilitated the development of effective vaccines for emergency use in a record time of less than one year. Nevertheless, since the end of 2020, a series of mutant variants have increased in prevalence globally, presumably due to selective pressure by adaptive immunity. Continued monitoring efforts of circulating and emerging variants will be vital to optimizing current and developing new effective vaccine and treatment approaches against HIV-1 and SARS-CoV-2. Both viruses unite in having a broad array of cellular anomalies and a pronounced cytokine storm correlated with disease severity. HIV-1 and SARS-CoV-2 co-infection must be considered an additional risk since an underlying immunodeficiency impairs SARS-CoV-2 clearance, which in turn may accelerate clinical aggravation and facilitate enhanced and prolonged viral evolution, and subsequent SARS-CoV-2 immune or drug escape.

## Figures and Tables

**Table 1:** Comparison of key features between SARS-CoV-2 and HIV-1 and their associated diseases.

	HIV-1	SARS-CoV-2	Refs
**Demographic features**			
geographic origin	West-Central Africa (Cameroon, DR Congo)	China (Wuhan)	[5,6]
first recorded case	HIV-1: 1959 (DR Congo)	SARS-CoV-2: Nov. 17, 2019 (China)	[7,8,9,10]
	AIDS: 1981 (USA)	COVID-19: Dec. 31, 2019 (China)	
est. time of origin/cross-species transmission	1920s	October/November 2019	[7,11,12,13]
animal source	non-human primates	primary host: bats, intermediate hosts: small mammals; yet unconfirmed	[5,14,15,16]
active cases	38 Mio ^a^	12 Mio ^b^	[17,18]
cases since pandemic start	76 Mio ^a^ (1.7 Mio new infections in 2019)	179 Mio ^b^	[17,18]
deaths since pandemic start	33 Mio ^a^ (0.7 Mio in 2019)	3.9 Mio ^b^	[17,18,19]
**Viral features**			
Baltimore virus classification	Group VI	Group IV	[20,21]
virus family	Retroviridae	Coronaviridae	
virus diameter	100–150 nm	60–140 nm	[6,22,23]
number of spikes per virus	7–14	15–40	[23,24,25]
spike size (height × width)	12 × 15 nm	20 × 13 nm	[26,27,28,29]
spike amino acids	856	1273	[30,31]
potential N-glyco sites per spike monomer	31 (HxB2)	22 (Wuhan-Hu-1)	[30,31]
spike proteolytic cleavage sites	1	2	[32,33]
capsid	Conical (many hexagons and 12 pentagons of subunits)	helical	[34,35,36]
genome	(+)ssRNA, diploid dsDNA genome intermediate	(+)ssRNA, haploid	[37,38]
genome size	9.7 kb (one of the smallest viral genomes)	29.7 kb (one of the largest viral genomes)	[30,31]
evolution rate	proviral DNA: 4 × 10^−3^ per base per cell (1 mutation every 250 base pairs)	1 × 10^−3^ per base per year (2 mutations per month)	[12,39,40]
	virus in plasma: 2–17 × 10^−3^ per base per year		
within-host diversity (in the absence of superinfection)	<5% (<10% for proviral env)	<0.05%	[41,42,43]
replication cycle	~24 h (in vitro)–60 h (in vivo)	~7–36 h	[44,45,46,47,48]
**Entry and host responses**			
primary target cells	CD4^+^ T cells, Macrophages	ACE2+ mucosal and endothelial cells	[49,50]
primary entry receptors/proteins	CD4, CCR5/CXCR4	ACE2, TMPRSS2	[51,52,53]
antibody response	Ab binding and neutralization response develops in first month	Ab binding and neutralization response develops in 1–2 weeks	[54,55,56,57]
	nAb development associated with viremia and severity	nAb development associated with viremia and severity	
	bnAb development usually requires 2–3 years of productive infection (observed in ~10% of HIV-1-infected individuals)	nAbs develop within weeks of infection	
	bnAbs require high rates of somatic hypermutation	Potent nAbs do not require high rates of somatic hypermutation (SHM), but SHM fosters breadth, potency, and resilience to viral escape	
cellular response	impaired B cell, T cell and macrophage/monocyte responses	impaired B cell, T cell and macrophage/monocyte responses	[58,59,60,61,62,63]
cytokine response	delayed and enhanced anti-inflammatory response, impaired IFN response in progressive cases	delayed and enhanced anti-inflammatory response, impaired IFN response in severe cases	[64,65,66,67,68,69,70]
**Disease features, treatment, and vaccines**			
clinical symptoms	AIDS	COVID-19	[6,49,71,72,73,74,75,76,77]
	(1) initially mild, common cold-like symptoms	(1) respiratory infection (fever, cough, sore throat, fatigue, loss of smell)	
	(2) acquired immune deficiency and opportunistic infections and malignancies	(2) systemic dissemination throughout the body (blood vessels, nervous system, inner organs)	
type of infection	chronic (HIV-1 integrates as provirus into host genome)	acute	
duration of infection	life-long	1–2 months (mild)	
		2–9 months (severe) and possible chronic complications	
primary site of infection	lymphatic system of gut and reproductive system	respiratory system	
primary mode of infection	sexual transmission	droplet infection of airways	
treatment	>45 FDA-approved drugs, strong viral-suppressive effect but no cure	(emergency use) authorization of a few drugs, limited clinical benefit (dexamethasone, remdesivir, nAb cocktails)	[70,78,79,80]
	drugs mainly target the polymerase region (reverse transcriptase, protease, and integrase)		
vaccine	no vaccine	(emergency use) authorization of a few vaccines, up to 95% vaccine efficacy	[81,82,83,84,85]
	7 vaccine efficacy trials completed, best efficacy: 31% (RV144, 2009)	>200 vaccine trials ongoing or completed	
correlates of protection	animal models: neutralizing antibodies;	neutralizing antibodies, supported by cellular responses	[81,86,87]
	human vaccine trial (RV144): ADCC, low plasma anti-Env IgA/IgG, poly-functional B cell responses, non-neutralizing V2 antibodies		

^a^ End of 2019; ^b^ June 2021.

**Table 2:** Amino acid replacements in global SARS-CoV-2 variants and their clinical impact.

Pangolin Lineage	B.1.1.7	B.1.351	P.1 (B.1.1.248)	B.1.617	B.1.1.298 (Cluster 5)	B.1.525	B.1.160	B.1.427 B.1.429	B.1.2	B.1.620	B.1.526
**Variant** **origin/first detected**	UK	South Africa	Brazil/Japan	India	Denmark (from minks)	Nigeria/UK	Europe	California	Midwest, USA	Cameroon, West-Central Africa/Lithuania, Europe	New York, NY, USA
**GISAID clade**	GRY	GH	GR	G	GR	G	GH	GH	GH	G	GH
**VOC/VOI**	VOC-20DEC-01	VOC-20DEC-02	VOC-21JAN-02	VOC-21APR-02 (B.1.617.2)	-	VOI VUI-21FEB-03	-	VOI	-	VOI	under monitoring-
**WHO (VOC/VOI)**	Alpha	Beta	Gamma	Delta (B.1.617.2)	-	Eta	-	Epsilon	-	-	Iota
**Other names/Nextstrain**	20I/S:501Y.V1	20H/S:501Y.V2	20J/S:501Y.V3	G/452R.V3 21A/S:478K	ΔFVI-Spike 20B	UK1188 20A/S:484K	20A.EU2-	CAL.20C20C /S:452R	COH.20G.Q677H -	20A	20C 20C/S:484K
**Clinical impact**	Epidemiological data suggest increased transmissibility and virulence; Little impact on vaccine efficacy	Suggested increased transmissibility but no influence on virulence; In vitro studies suggest partial **nAb immune escape and reduced vaccine efficacy**	Effect on transmissibility and virulence under investigation; In vitro studies suggest partial **nAb immune escape and reduced vaccine efficacy**	Epidemiological data suggest increased transmissibility; In vitro studies suggest partial **nAb immune escape and reduced vaccine efficacy**	Suggested increased transmissibility; no evidence of increased virulence or vaccine immune evasion	Suggested to have partial **nAb immune escape and reduced vaccine efficacy**	No evidence of increased transmissibility, virulence, or immune evasion	Epidemiological data suggest increased transmissibility; In vivo and pseudovirus data suggest increased virulence and partial immune evasion	No evidence of increased transmissibility, virulence, or immune evasion	Suggested to have partial **nAb immune escape and reduced vaccine efficacy**	Suggested to have increased transmissibility; no evidence of increased virulence yet; partial **nAb immune escape and reduced vaccine efficacy** predicted
**Amino acid mutations and deletions**											
**NSP1**	-	-	-	-	Δ M85	-	-	-	-	-	-
**NSP2**	-	**T85I**	-	-	-	-	-	**T85I** ^#^	**T85I**	T223I	**T85I**
**NSP3 (PL-pro)**	T183I, A890D, I1412T	K837N	S370L, K977Q	-	Δ N1264	T1189I	-	-	M1788I	V1173I	-
**NSP4**	-	H36Y, S137L	-	-	-	-	M324I	-	-	-	L438P
**NSP5 (3CL-pro)**	-	K90R	-	-	-	-	-	-	L89F	-	-
**NSP6**	**ΔS106-G107-F108**	-	**ΔS106-G107-F108**	-	-	**ΔS106-G107-F108**	-	-	-	**ΔS106-G107-F108**	**ΔS106-G107-F108**
**NSP9**	-	-	-	-	-	-	-	I65V ^#^	-	-	-
**NSP12 (RdRp)**	**P323L**	D144Y, **P323L**	**P323L**	**P323L**	**P323L**, T739I	P323F*	A185S, **P323L**, V776L	**P323L**	**P323L**	**P323L**	**P323L**
**NSP13 (Helicase)**	-	T588I	E341D	P77L ^#^, M429I ^#^	-	-	K218R, E261D	P53L ^#^, D260Y	-	A292S	Q88H
**NSP14**	-	-	-	-	-	-	-	-	N129D	-	-
**NSP15**	-	-	-	K259R ^#^	T112I	-	-	-	-	-	-
**NSP16**	-	-	-	-	-	-	-	-	R216C	-	-
**Spike**	**ΔH69–V70, ΔY144, N501Y**, A570D, **D614G, P681H**, T716I S982A, **D1118H**	**L18F,** D80A D215G, **ΔL241-L242-A243**, R246I, K417N *, E484K, N501Y **D614G, A701V**	**L18F**, T20N, **P26S**, D138Y, R190S, K417T *, E484K, N501Y, D614G, H655Y, **T1027I**, V1176F	G142D ^#^, E154K ^#^, **L452R**, T478K ^#^, **E484Q** ^#^*, **D614G, P681R**	**ΔH69–V70**, Y453F, **D614G** I692V, M1229I	Q52R, A67V, **ΔH69–V70**, **ΔY144**, **E484K, D614G, Q677H**, F888L	**S477N**, **D614G**	S13I, W152C, **L452R**, **D614G**	**D614G, Q677H**	**P26S**, **ΔH69–V70**, V126A, **ΔY144**, **ΔL241-L242-A243**, H245Y, **S477N, E484K, D614G, P681H, T1027I, D1118H**	L5F ^#^, T95I, D253G, **L452R** ^#^, **S477N** ^#^, **E484K** ^#^, **D614G, A701V** ^#^, Q957R ^#^
**ORF3a**	-	**Q57H**, S171L	S253P	S26L ^#^	H182Y	-	**Q57H**	**Q57H**	**Q57H**, G172V	-	P42L, **Q57H**
**M**	-	-	-	**I82T/S** ^#^	-	**I82T**	-	-	A85S	-	-
**ORF7a**	-	-	-	V82A	-	-	-	-	-	-	-
**ORF8**	Q27stop, R52I, Y73C	-	E92K	-	-	-	-	-	S24L	-	T11I
**ORF9b**	-	-	-	-	-	-	-	-	-	I5T	-
**E**	-	P71L	-	-	-	L21F	-	-	-		-
**N**	D3L, **R203K, G204R**, S235F	**T205I**	P80R, **R203K, G204R**	R203M * D377Y	S194L, **R203K, G204R**	A12G, **T205I**	**M234I**, A376T	**T205I**	P67S, **P199L, D377Y**	A220V	**P199L**^#^, **M234I**^#^

Bold type: Mutation found in multiple variants listed. * Mutation at the same amino acid position found in other variants but with another amino acid replacement. ^#^ Mutation found in subsets of the variant. VOC: variant of concern; VOI: variant of interest.

## Data Availability

Analyses were performed using publicly archived datasets as indicated. Protein structures were generated using pdb files downloaded from the RCSB Protein Data Bank (RCSB PDB; https://www.rcsb.org/), the Electron Microscopy Data Bank (EMDB; https://www.ebi.ac.uk/pdbe/emdb/), and the Zhang lab webpage on COVID-19 structure modeling (https://zhanglab.ccmb.med.umich.edu/COVID-19/). Viral sequences were downloaded from GISAID (https://www.gisaid.org/) and the LANL HIV Databases (https://www.hiv.lanl.gov/content/index).
